# Hepatocyte‐Specific GSDMD Deficiency Aggravates Sepsis by Disrupting Non‐Canonical Secretion of Anti‐Inflammatory Factors

**DOI:** 10.1002/advs.202510412

**Published:** 2025-08-26

**Authors:** Yihan Qian, Bingrui Wang, Chang Yu, Yuge Zhou, Weifan Huang, Xing Rong, Yali Sang, Jiangang Song, Hailong Wu, Xiaoni Kong

**Affiliations:** ^1^ Central Laboratory Shuguang Hospital Affiliated to Shanghai University of Traditional Chinese Medicine Shanghai 201203 China; ^2^ Shanghai Key Laboratory of Molecular Imaging Collaborative Innovation Center for Biomedicines Shanghai University of Medicine and Health Sciences Shanghai 201318 China; ^3^ Department of anaesthesiology Shuguang Hospital Affiliated to Shanghai University of Traditional Chinese Medicine Shanghai 201203 China; ^4^ KELUN‐BIOTECH Shanghai 200023 China

**Keywords:** anti‐inflammatory factors, gasdermin D, hepatocyte, macrophage, sepsis

## Abstract

Gasdermin D (GSDMD)‐mediated pyroptosis in macrophages plays a clear role in promoting inflammation and mortality in sepsis. The liver is a commonly damaged organ during sepsis and also an important organ for releasing acute response proteins. However, whether pyroptosis occurs and the function of GSDMD in hepatocytes remains unclear. It is surprising to find that hepatocyte‐specific GSDMD knockout (GSDMD^hep‐/‐^) mice have significantly reduced survival rates, markedly elevated systemic inflammation, and increased inflammation in the peritoneal cavity and lungs, suggesting that the absence of GSDMD in hepatocytes promotes systemic inflammatory responses. Serum proteomic analysis shows that anti‐inflammatory factors such as VEGF‐B and Gremlin‐1 are significantly reduced in GSDMD^hep‐/‐^ mice. Through in vitro and in vivo experiments combined with a constructed full‐length GSDMD and a mutant GSDMD plasmid (GSDMD‐c.D276A) that cannot be cleaved, VEGF‐B and Gremlin‐1 are verified to be released from hepatocytes through the pore‐forming activity of GSDMD, thus inhibiting the production of inflammatory factors by macrophages. More importantly, hepatocyte‐specific replenishment of full‐length GSDMD can reverse the exacerbated inflammatory response in GSDMD^hep‐/‐^ mice. These findings together establish that hepatic GSDMD plays a key protective role in sepsis by promoting the release of anti‐inflammatory factors through pore formation in hepatocytes.

## Graphical abstract

This study proposes for the first time that hepatic GSDMD plays a key protective role in sepsis. Hepatic GSDMD may release inflammatory inhibitory factors including VEGF‐B and Gremlin‐1 through pore‐forming activity to inhibit the production of inflammatory factors from macrophages, thereby alleviating the inflammatory response of septic mice.

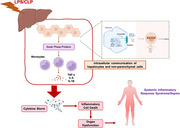

## 1 Introduction

Sepsis is characterized by life‐threatening organ dysfunction due to a dysregulated host response to infection.^[^ ^1^ ^]^ The liver plays a central role during sepsis and is essential for regulating immune defense during systemic infection through mechanisms such as bacterial clearance, acute phase protein or cytokine production, and metabolic adaptation to inflammation.^[^ ^2^ ^]^

In patients with sepsis, macrophage pyroptosis and the release of inflammatory factors by macrophages are mainly related to the immune response and activation of other immune cells. However, as an organ which is damaged in sepsis, the liver has been proven to play an important role in sepsis.^[^ ^2^, ^3^ ^]^ However, whether hepatocytes undergo pyroptosis in sepsis and the role of hepatocyte pyroptosis in sepsis remain unclear. As a new type of proinflammatory programmed cell death, pyroptosis is an inflammasome and caspase‐1‐dependent form of necrosis. Activated caspase‐1 leads to the cleavage of the pyroptosis‐inducing factor Gasdermin D (GSDMD) by producing IL‐1β and IL‐18. The release of IL‐1β and IL‐18 can lead to local and systemic inflammation of cells, ultimately leading to cell death cells, which are characterized by the activation of two different types of caspase enzymes and the occurrence of proinflammatory cytokine cascades and immune responses.^[^ ^4^ ^]^ Excessive cell pyroptosis can lead to the development of liver disease, while excessive inflammatory responses can release liver danger signals, thereby activating inflammasomes and other cell death mechanisms.^[^ ^5^ ^]^

Since the Gasdermin gene family was discovered in the early 21st century, it has been believed to play a negative role in various diseases by inducing pyroptosis and causing cell death. As a typical member of the Gasdermin gene family, GSDMD contains a 31 kDa cytotoxic N‐terminal domain and a 22 kDa C‐terminal domain. A variety of cellular proteases can cleave GSDMD and activate its pore‐forming activity. Cytosolic lipopolysaccharide (LPS) can be sensed by caspase‐11. And inflammasome activation can activate caspase‐1, while TNF receptor signaling activates caspase‐8. These proteases can cleave GSDMD within the interdomain linker which connects the C‐terminal domain and the N‐terminal domain of GSDMD.^[^ ^6^ ^]^ The N‐terminal domain enables GSDMD to target, insert and destroy cell membranes by forming membrane pores, and protein hydrolysis between the two domains releases the intramolecular inhibition of the cytotoxic domain into the cell membrane and forms large oligomeric pores, destroying ion homeostasis, thereby destroying membrane integrity, inducing cell damage and inflammatory cytokine release.^[^ ^7^, ^8^ ^]^

Studies have proved that in sepsis, the dysregulation of the inflammatory response ultimately leads to a fatal inflammatory storm, and pyroptosis plays an important role in sepsis. During pyroptosis, immune cells release proinflammatory cytokines to recruit other immune cells to fight infection, thus enhancing the defense response and promoting the elimination of invading pathogens.^[^ ^9^ ^]^ As an important natural immune response, pyroptosis helps to resist infection and endogenous danger signals. However, excessive activation of this process is the main cause of immune dysregulation in sepsis. A large number of immune cells pyroptosis in a short period of time can cause a strong inflammatory storm and organ dysfunction, as well as immune cell failure.^[^ ^10^ ^]^ As the executor of pyroptosis, GSDMD can regulate organ damage, cell regulation, and molecular regulation that play a leading role in the occurrence and development of sepsis. In the LPS‐induced sepsis model, caspase‐11 and GSDMD knockout mice (GSDMD^−/−^ mice) showed significantly improved survival rates.^[^ ^11^ ^]^ Mechanistically, activation of the caspase‐11/GSDMD pathway controls the neutrophil extracellular trap (NET) release from neutrophils during sepsis.^[^ ^12^ ^]^ Inhibition of GSDMD with disulfiram or genetic deletion can eliminate excessive NET formation, thereby reducing organ dysfunction and mortality in sepsis.

However, in recent years, some studies have found that GSDMD‐mediated cell pore‐forming activity and pyroptosis can play a repair role in some cases. Mehrotra et al. believed that the dominant role of IL‐1β in promoting tissue inflammation in pyroptosis masked the potential effects of other factors released by pyroptosis. They designed a system to induce pyroptosis in macrophages without releasing IL‐1β or IL‐1α, and the results showed that its supernatant could promote faster wound closure in vitro and improve tissue repair in vivo. This effect was related to the synthesis of oxylipin prostaglandin E2 (PGE2), which was synthesized in the late stage of pyroptosis, and its release depended on the pore‐forming activity of GSDMD during pyroptosis.^[^ ^13^ ^]^ In addition, Chi et al. identified 11,12‐epoxyeicosatrienoic acid (11,12‐EET), a bioactive pro‐healing oxidized lipid, was secreted from overactive macrophages in a GSDMD‐dependent manner, further demonstrating that macrophage GSDMD might play a positive role in tissue recovery.^[^ ^14^ ^]^

The specific role of GSDMD in hepatocytes is still unclear. Therefore, in this study, we constructed hepatocyte‐specific GSDMD knockout mice (GSDMD^hep‐/−^) and control mice (GSDMD^flox+/+^) to reveal the role of hepatic GSDMD in sepsis. Contrary to previous studies that GSDMD plays a negative role in sepsis, we found that liver‐specific knockout of GSDMD aggravated inflammatory responses and lung injury in both LPS‐ and CLP‐induced sepsis model. To further explore the mechanism of GSDMD in hepatocytes in inhibiting inflammation response in sepsis, we performed serum proteomic analysis and found that anti‐inflammatory factors such as VEGF‐B and Gremlin‐1 were significantly reduced in GSDMD^hep‐/−^ mice. Further in vitro and in vivo experiments verified that anti‐inflammatory factors VEGF‐B and Gremlin‐1 were released from hepatocytes through the pore‐forming activity of GSDMD. And these anti‐inflammatory factors to inhibit the production of inflammatory factors by macrophages. More importantly, hepatocyte‐specific replenishment of full‐length GSDMD could reverse the exacerbated inflammatory response in GSDMD^hep‐/−^ mice, further indicating that hepatic GSDMD might play a protective role in sepsis by promoting the release of anti‐inflammatory factors through pore formation in hepatocytes.

## 2 Results

### 2.1 Pyroptosis can be Observed in Liver/Hepatocytes in the Sepsis Model

**Figure 1:**
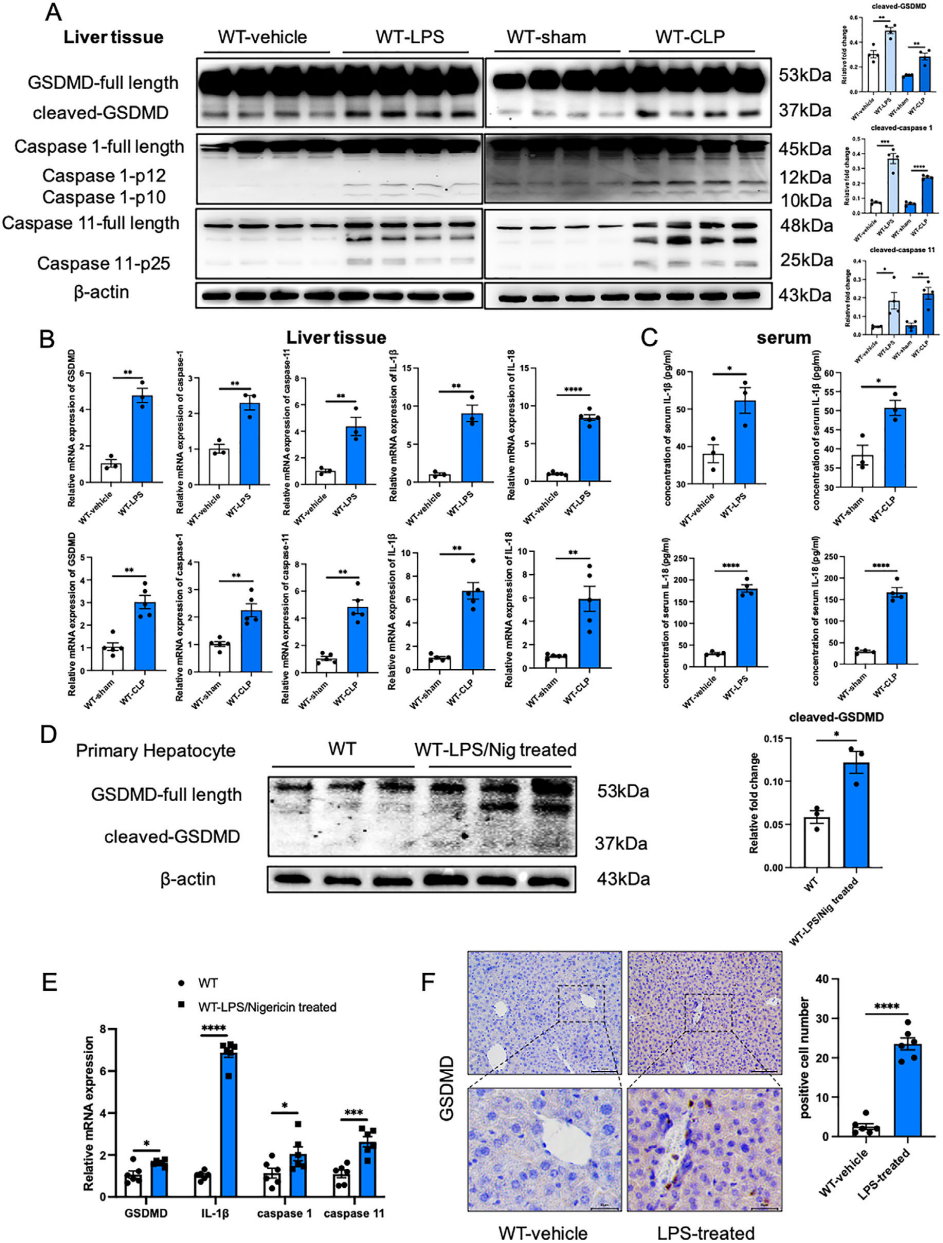
Pyroptosis occurs in the liver of septic mice, and the expression of GSDMD in the liver tissue of septic mice is significantly increased. A) Protein levels of pyroptosis‐related proteins (GSDMD, caspase‐1, caspase‐11) and their spliceosomes in the liver tissue of septic mice induced by LPS and CLP (*n* = 4). B) mRNA levels of GSDMD, caspase‐1, caspase‐11, IL‐1β, and IL‐18 in the liver tissues of septic mice (*n* = 3–5). C) IL‐1β and IL‐18 content in the serum of septic mice (*n* = 3–4). D) Protein levels of GSDMD and its spliceosome in the in vitro pyroptosis model of primary mouse hepatocytes (*n* = 3). E) mRNA levels of GSDMD, caspase‐1, caspase‐11, and IL‐1β in the in vitro pyroptosis model of primary mouse hepatocytes (n = 6). F) GSDMD staining in the liver tissue of septic mice (*n* = 6). Data are presented as the mean ± SEM, ^*^ *p* <0.05, ^**^ *p* <0.01, ^***^ *p* <0.001, ^****^ *p* <0.001.

First, we identified whether pyroptosis could be observed in the liver of septic mice. Our results demonstrated that pyroptosis occurred in the liver in the sepsis model induced by both LPS and CLP, evidenced by a significant increase in the expression levels of cleaved‐GSDMD, cleaved‐caspase‐1, and cleaved‐caspase‐11 (**Figure** **1**A). In addition, qPCR results showed that the mRNA levels of GSDMD, IL‐1β, caspase‐1, and caspase‐11 in liver tissues were significantly increased in LPS‐ and CLP‐induced sepsis models, and the serum concentration of IL‐1β in the sepsis model was also significantly increased (Figure 1B,C). The occurrence of pyroptosis in the sepsis model was also reflected in the significantly enhanced GSDMD‐positive cell staining in mouse liver tissues (Figure 1F). Furthermore, we also extracted mouse primary hepatocytes and induced a pyroptosis model in vitro, and the results showed consistent conclusions with those in liver tissue (Figure 1D,E).

### 2.2 Hepatic GSDMD Deficient Mice are more Susceptible to Both LPS‐ and CLP‐Induced Sepsis and Induce more Severe Systemic Inflammatory Reactions

**Figure 2:**
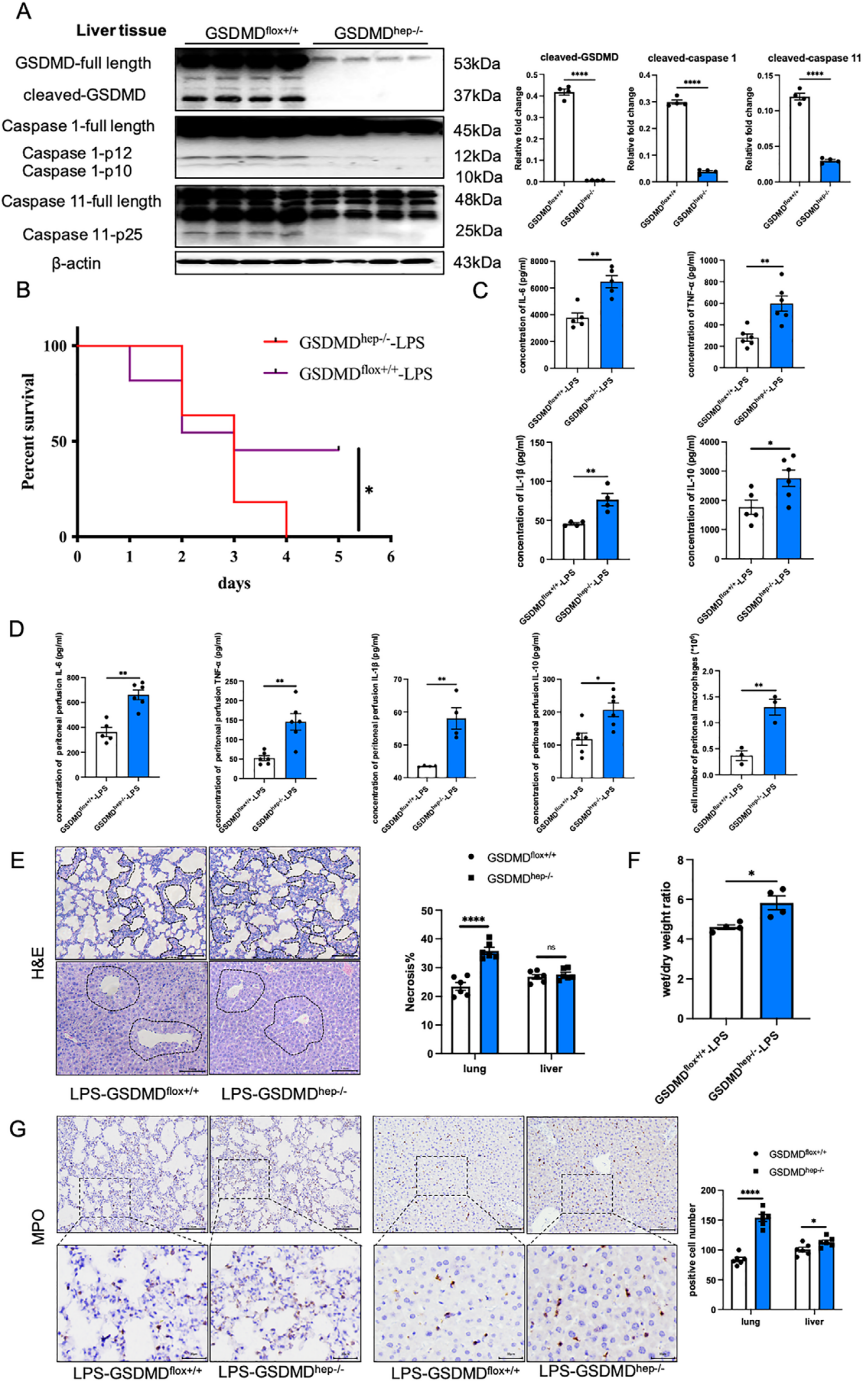
Liver‐specific knockout of GSDMD significantly reduced the survival rate of LPS‐induced septic mice and aggravated inflammation and lung injury. A) Protein levels of pyroptosis‐related proteins and their spliceosomes in LPS‐induced sepsis models of GSDMD^flox+/+^ mice and GSDMD^hep‐/−^ mice (*n* = 4). B) Survival rate of mice in LPS‐induced sepsis models of GSDMD^flox+/+^ mice and GSDMD^hep‐/−^ mice (*n* = 10). C) Contents of inflammatory factors in the serum of LPS‐induced sepsis models of GSDMD^flox+/+^ mice and GSDMD^hep‐/−^ mice (*n* = 4–6). D) Contents of inflammatory factors in peritoneal perfusion of LPS‐induced sepsis models of GSDMD^flox+/+^ mice and GSDMD^hep‐/−^ mice (*n* = 4–6). E) H&E staining of lung and liver tissues in LPS‐induced sepsis models of GSDMD^flox+/+^ mice and GSDMD^hep‐/−^ mice (*n* = 6). F) The lung wet‐to‐dry weight ratio of LPS‐induced sepsis models of GSDMD^flox+/+^ mice and GSDMD^hep‐/−^ mice (*n* = 4). G) MPO staining of lung and liver tissues in LPS‐induced sepsis models of GSDMD^flox+/+^ mice and GSDMD^hep‐/−^ mice (*n* = 6). Data are presented as the mean ± SEM, ^*^ *p* <0.05, ^**^ *p* <0.01, ^****^ *p* <0.001.

In order to investigate the role of hepatic GSDMD in sepsis, we constructed GSDMD^flox+/+^ mice and generated liver‐specific knockout GSDMD mice (GSDMD^hep‐/−^ mice) using GSDMD^flox+/+^ mice and Alb‐Cre mice (Figure S1, Supporting Information). Western blotting results showed that pyroptosis almost did not occur in the liver tissue of GSDMD^hep‐/−^ mice (**Figure** **2**A). However, in contrast to the results of GSDMD^−/−^ mice, the survival rate of GSDMD^hep‐/−^ mice was surprisingly lower in the LPS‐induced sepsis model (Figure 2B). The ELISA results also showed that the levels of inflammatory factors in the serum and peritoneal perfusion of GSDMD^hep‐/−^ mice were significantly increased, and the number of peritoneal macrophages was also significantly higher in GSDMD^hep‐/−^ mice, indicating that liver‐specific knockout of GSDMD would aggravate the inflammatory response of septic mice (Figure 2C,D). The lung wet‐to‐dry weight ratio also showed that the wet/dry weight ratios of GSDMD^hep‐/−^ mice were significantly higher than those of GSDMD^flox+/+^ mice (Figure 2F), indicating that hepatic GSDMD deficient mice were more susceptible to sepsis. We then detected the H&E staining and the MPO staining of mouse liver and lung tissue sections. The results showed that liver‐specific knockout of GSDMD significantly exacerbated lung injury in LPS‐induced septic, while having relatively little effect on liver injury (Figure 2E,G). The ALT/AST analysis also proved that hepatic GSDMD deficient did not affect the dysfunction of liver in sepsis (Figure S4, Supporting Information).

**Figure 3:**
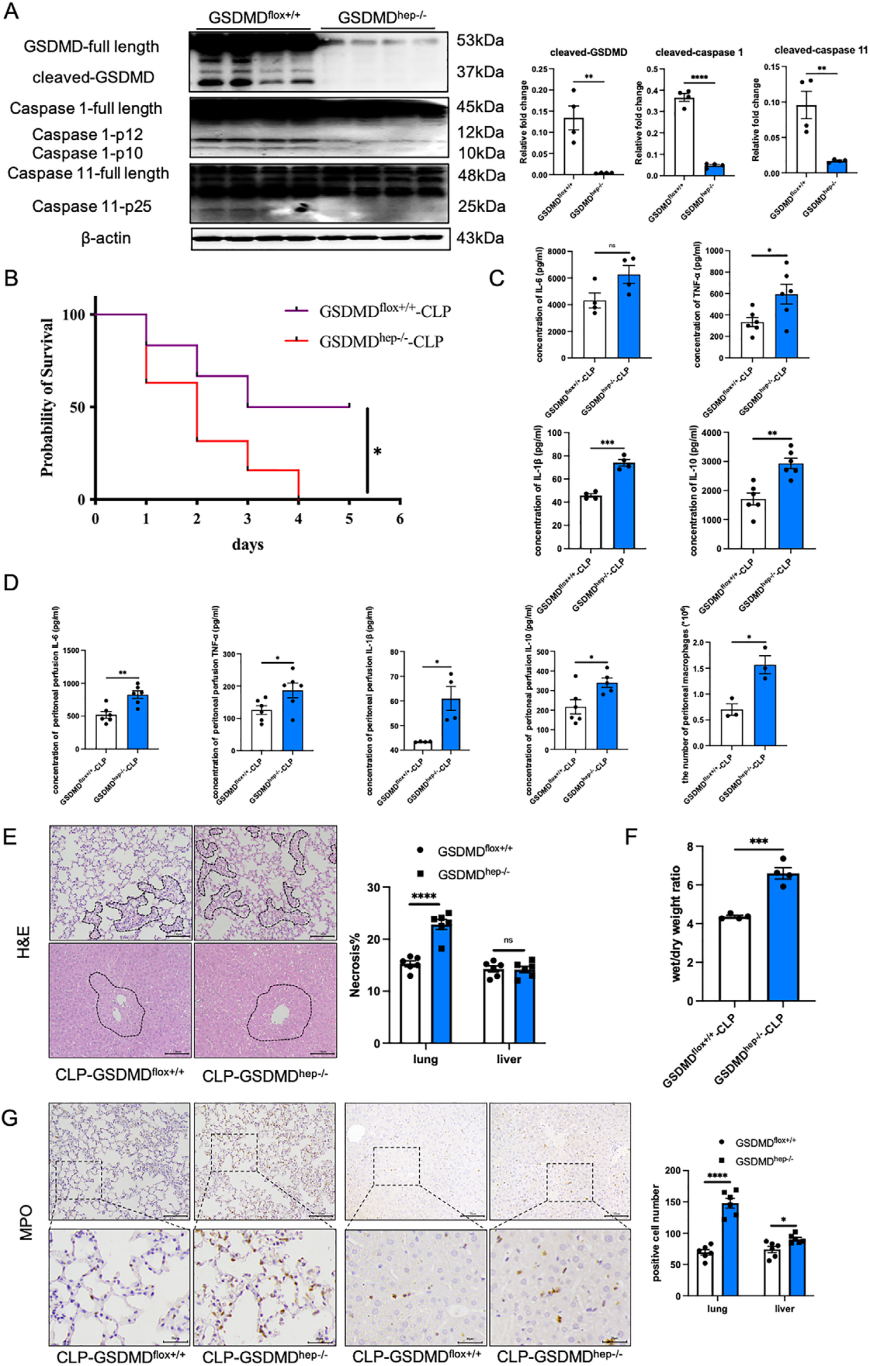
Liver‐specific knockout of GSDMD significantly reduced the survival rate of CLP‐induced septic mice and aggravated inflammation and lung injury. A) Protein levels of pyroptosis‐related proteins and their spliceosomes in CLP‐induced sepsis models of GSDMD^flox+/+^ mice and GSDMD^hep‐/−^ mice (*n* = 4). B) Survival rate of mice in CLP‐induced sepsis models of GSDMD^flox+/+^ mice and GSDMD^hep‐/−^ mice (*n* = 10). C) Contents of inflammatory factors in the serum of CLP‐induced sepsis models of GSDMD^flox+/+^ mice and GSDMD^hep‐/−^ mice (*n* = 4–6). D) Contents of inflammatory factors in peritoneal perfusion of CLP‐induced sepsis models of GSDMD^flox+/+^ mice and GSDMD^hep‐/−^ mice (*n* = 4–6). E) H&E staining of lung and liver tissues in CLP‐induced sepsis models of GSDMD^flox+/+^ mice and GSDMD^hep‐/−^ mice (*n* = 6). F) The lung wet‐to‐dry weight ratio of CLP‐induced sepsis models of GSDMD^flox+/+^ mice and GSDMD^hep‐/−^ mice (*n* = 4). G) MPO staining of lung and liver tissues in CLP‐induced sepsis models of GSDMD^flox+/+^ mice and GSDMD^hep‐/−^ mice (*n* = 6). Data are presented as the mean ± SEM, ^*^ *p* <0.05, ^**^ *p* <0.01, ^***^ *p* <0.001, ^****^p <0.001.

To further validate our results, we repeated the above experiments in the cecal ligation and puncture (CLP)‐induced sepsis model, which closely resembles the progression and characteristics of human sepsis.^[^ ^15^ ^]^ The results showed that liver‐specific knockout of GSDMD significantly aggravated the inflammatory response and lung injury in CLP‐induced sepsis, which was consistent with the results of the LPS‐induced sepsis model (**Figure** **3**). These results confirmed that hepatic GSDMD deficient mice were more susceptible to both LPS‐ and CLP‐induced sepsis and induced more severe systemic inflammatory reactions.

### 2.3 Hepatic GSDMD Deficient Mice Show Lower Expression Levels of Anti‐Inflammatory Factors Screened by Serum Proteomics

**Figure 4:**
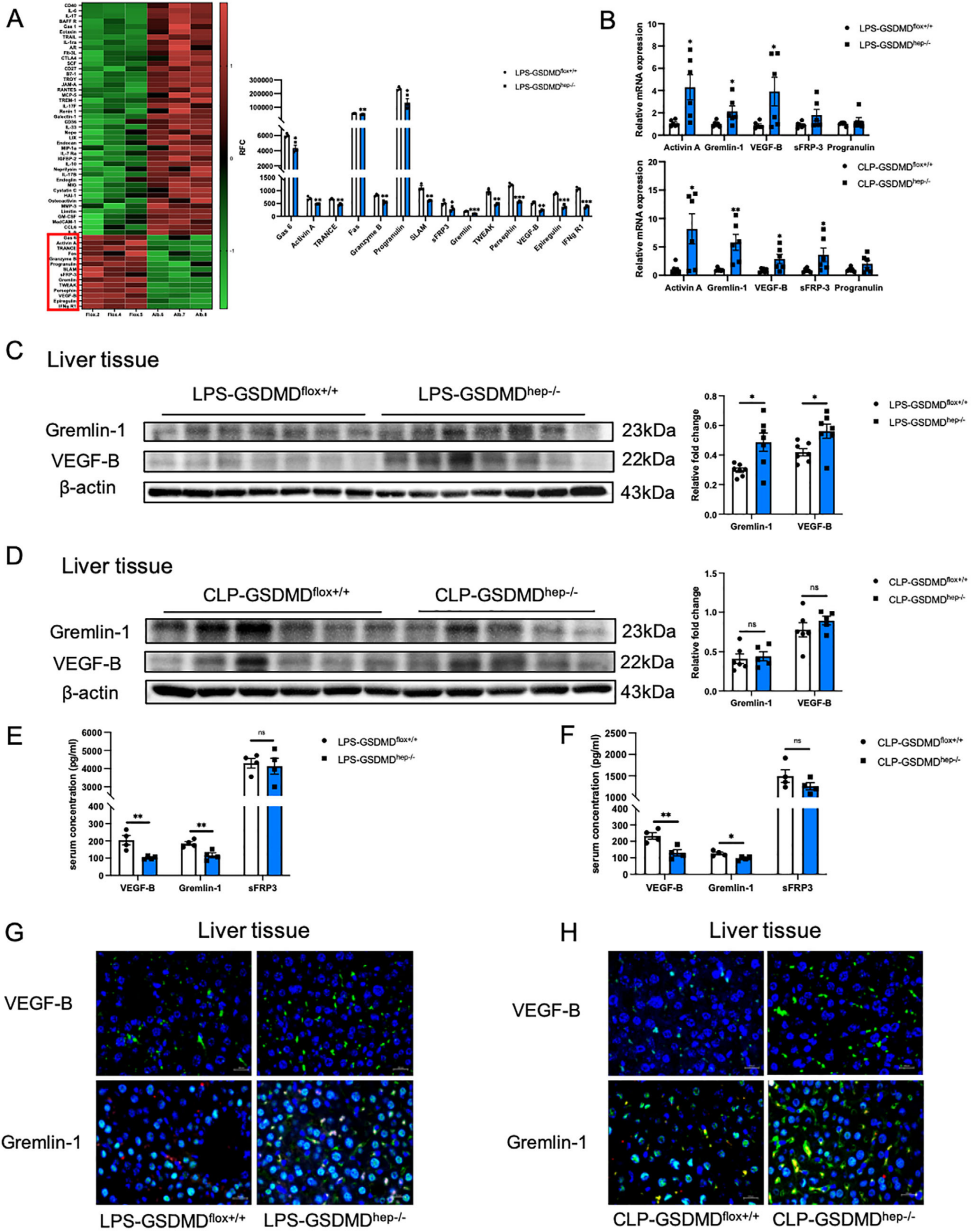
Liver‐specific knockout of GSDMD decreased the expression levels of anti‐inflammatory factors screened by serum proteomics. A) Serum proteomics of LPS‐induced sepsis models of GSDMD^flox+/+^ mice and GSDMD^hep‐/−^ mice. Proteins with significantly reduced levels in the serum of GSDMD^hep‐/−^ mice are marked by red boxes (*n* = 3). B) mRNA levels of Activin A, Gremlin‐1, VEGF‐B, sFRP‐3 and Progranulin in the serum of LPS‐ and CLP‐induced sepsis models of GSDMD^flox+/+^ mice and GSDMD^hep‐/−^ mice (*n* = 6). C) Protein levels of Gremlin‐1 and VEGF‐B in the liver tissues of LPS‐induced sepsis models of GSDMD^flox+/+^ mice and GSDMD^hep‐/−^ mice (*n* = 7). D) Protein levels of Gremlin‐1 and VEGF‐B in the liver tissues of CLP‐induced sepsis models of GSDMD^flox+/+^ mice and GSDMD^hep‐/−^ mice (*n* = 5–6). E) Contents of Gremlin‐1, VEGF‐B and sFRP‐3 in the serum of LPS‐induced sepsis models of GSDMD^flox+/+^ mice and GSDMD^hep‐/−^ mice (*n* = 4). F) Contents of Gremlin‐1, VEGF‐B and sFRP‐3 in the serum of CLP‐induced sepsis models of GSDMD^flox+/+^ mice and GSDMD^hep‐/−^ mice (*n* = 4). G) Immunofluorescent staining of Gremlin‐1 and VEGF‐B in the liver tissues of LPS‐induced sepsis models of GSDMD^flox+/+^ mice and GSDMD^hep‐/−^ mice (Blue: DAPI, Green: VEGF‐B; Blue: DAPI, Green: HNF4α, Red: Gremlin‐1). H) Immunofluorescent staining of Gremlin‐1 and VEGF‐B in the liver tissues of CLP‐induced sepsis models of GSDMD^flox+/+^ mice and GSDMD^hep‐/−^ mice (Blue: DAPI, Green: VEGF‐B; Blue: DAPI, Green: HNF4α, Red: Gremlin‐1). Data are presented as the mean ± SEM, ^*^ *p* <0.05, ^**^ *p* <0.01, ^***^p <0.001.

Later, we performed serum proteomic analysis on the serum of mice in the LPS‐induced sepsis models of GSDMD^flox+/+^ mice and GSDMD^hep‐/−^ mice and found that the levels of anti‐inflammatory factors (Gas6, Activin A, TRANCE, Fas, sFRP3, Gremlin‐1, TWEAK, VEGF‐B, etc.) in the serum of GSDMD^hep‐/−^ mice were significantly reduced (**Figure** **4**A). According to the GO analysis, the differences between LPS‐treated GSDMD^flox+/+^ mice and GSDMD^hep‐/−^ mice were mainly enriched in cytokine‐mediated signaling pathway, cellular response to interleukin‐1, chemoattractant activity, and vascular endothelial growth factor receptor 2/3 binding. The KEGG analysis results suggested that the differences between the two groups were mainly enriched in pertussis and TNF signaling pathway (Figure S5, Supporting Information), demonstrating that hepatic GSDMD might play a role in the inflammatory response mediated by cytokines and secretory proteins in sepsis.

Through qPCR analysis of liver tissues and ELISA detection of mouse serum, we finally screened out two anti‐inflammatory factors, Gremlin‐1 and VEGF‐B, for subsequent experiments (Figure 4B,E). The results of western blotting on liver tissues and immunofluorescence on liver tissue sections showed that in the LPS‐ and CLP‐induced sepsis models, the expression levels of Gremlin‐1 and VEGF‐B in the liver tissue of GSDMD^hep‐/−^ mice were higher than those of GSDMD^flox+/+^ mice (Figure 4D,G). Based on the cell membrane pore‐forming effect of GSDMD, we speculated that hepatic GSDMD might release Gremlin‐1 and VEGF‐B from hepatocytes into the serum through cell membrane pores, thereby alleviating sepsis.

### 2.4 GSDMD Plays its Role in LPS and CLP Model in a Hepatocyte Membrane Pores‐Dependent Manner In Vitro

**Figure 5:**
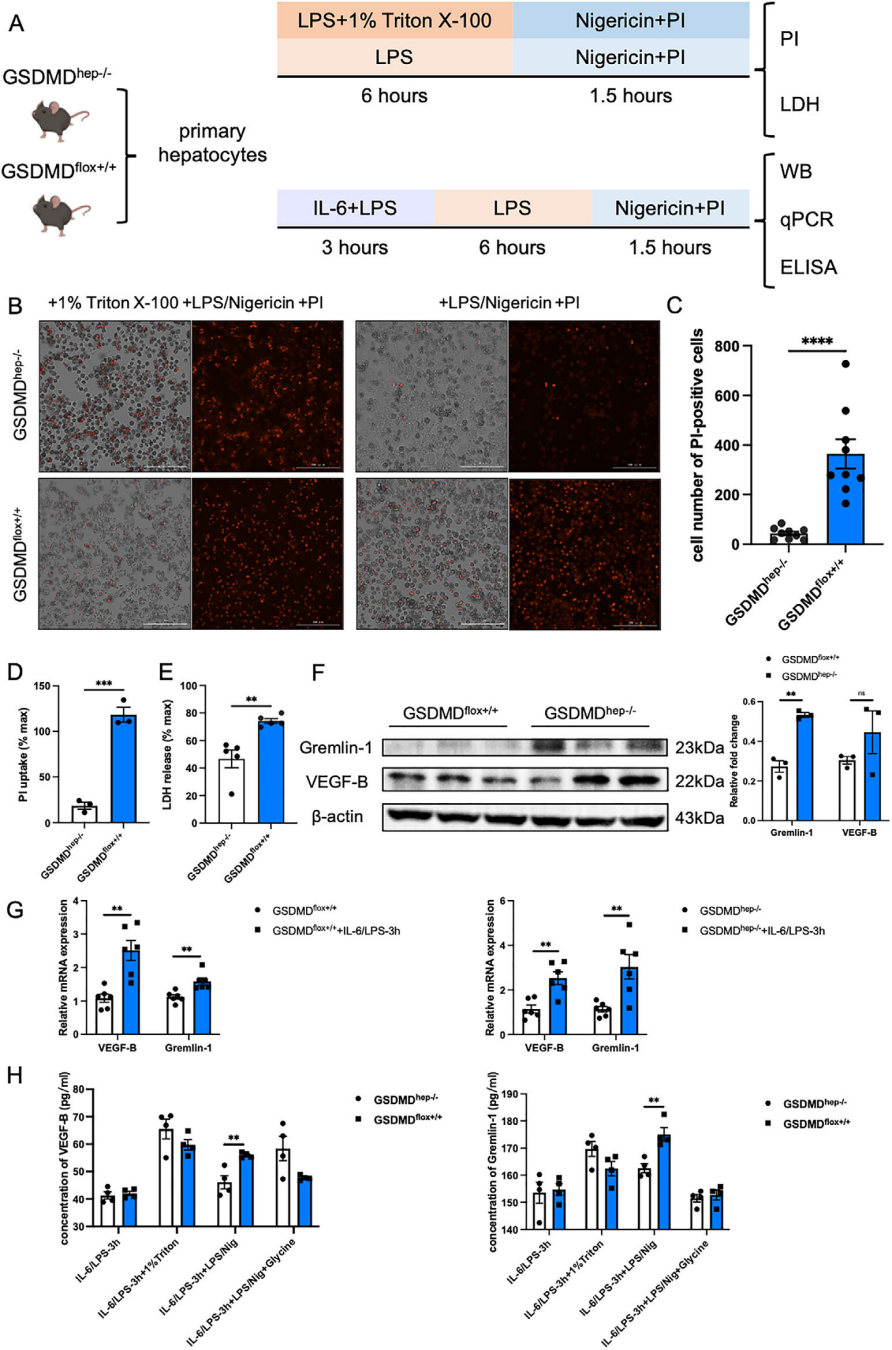
GSDMD played its role in the LPS and CLP model in a hepatocyte membrane pores‐dependent manner. A) Schematic diagram of in vitro experiments on primary hepatocytes. B) PI staining in LPS/Nigericin‐treated primary hepatocytes derived from GSDMD^flox+/+^ mice and GSDMD^hep‐/−^ mice. C,D) Cell number of PI‐positive cells (*n* = 9) and PI uptake (*n* = 3) in Figure B. E) LDH release of LPS/Nigericin‐treated primary hepatocytes derived from GSDMD^flox+/+^ mice and GSDMD^hep‐/−^ mice (*n* = 5). F) Protein levels of Gremlin‐1 and VEGF‐B in LPS/Nigericin‐treated primary hepatocytes derived from GSDMD^flox+/+^ mice and GSDMD^hep‐/−^ mice (*n* = 3). G) mRNA expression of VEGF‐B and Gremlin‐1 in LPS/Nigericin‐treated primary hepatocytes derived from GSDMD^flox+/+^ mice and GSDMD^hep‐/−^ mice after 3 h treatment of IL‐6 and LPS (*n* = 6). H) Concentration of cell culture supernatant VEGF‐B and Gremlin‐1 in LPS/Nigericin‐treated primary hepatocytes derived from GSDMD^flox+/+^ mice and GSDMD^hep‐/−^ mice after 3 h treatment of IL‐6 and LPS (*n* = 4). Data are presented as the mean ± SEM, ^**^ *p* <0.01, ^***^p <0.001, ^****^ *p* <0.001.

In LPS‐ and CLP‐induced sepsis models, we found that hepatic GSDMD did not exacerbate liver injury in septic mice, as well as the dysfunction of the liver (Figure S4, Supporting Information), so we speculated that in addition to playing a pyroptotic role, hepatic GSDMD played other roles in sepsis, such as pore‐forming activity. In order to verify whether the role of hepatic GSDMD in sepsis depended on its cell membrane pore formation, we established the in vitro cell pyroptosis model in mouse primary hepatocytes (1 µg mL^−1^ LPS treatment for 6 h and then 20 µm Nigericin stimulation for 1.5 h) (**Figure** **5**A). The number of PI‐positive cells and PI uptake showed that hepatocytes from GSDMD^hep‐/−^ mice almost did not form cell membrane pores, which was also confirmed by LDH release detection results (Figure 5B–E). Subsequently, we tried to verify the role of Gremlin‐1 and VEGF‐B in the cell pyroptosis model in vitro and found that compared with the complex environment in vivo, simple LPS/Nigericin stimulation could not activate the expression and release of Gremlin‐1 and VEGF‐B in primary hepatocytes. Therefore, we used various combinations of inflammatory factors to try to activate the secretion of Gremlin‐1 and VEGF‐B (Figure S6, Supporting Information). The screening results suggested that stimulating primary hepatocytes with 20ng mL^−1^ IL‐6 and 1µg mL^−1^ LPS for 3 h and then inducing pyroptosis with LPS/Nigericin could significantly increase the mRNA and protein expression levels of Gremlin‐1 and VEGF‐B in primary hepatocytes (Figure 5F,G). ELISA detection of cell supernatants also confirmed that primary hepatocytes of GSDMD^hep‐/−^ mice reduced the release of Gremlin‐1 and VEGF‐B from hepatocytes (Figure 5H), indicating that the release of Gremlin‐1 and VEGF‐B from hepatocytes depended on the cell membrane pore‐forming activity of GSDMD in hepatocytes.

### 2.5 GSDMD Induces the Release of Anti‐Inflammatory Factors in Primary Hepatocytes Derived from GSDMDhep‐/− Mice through its Pore‐Forming Activity In Vitro

**Figure 6:**
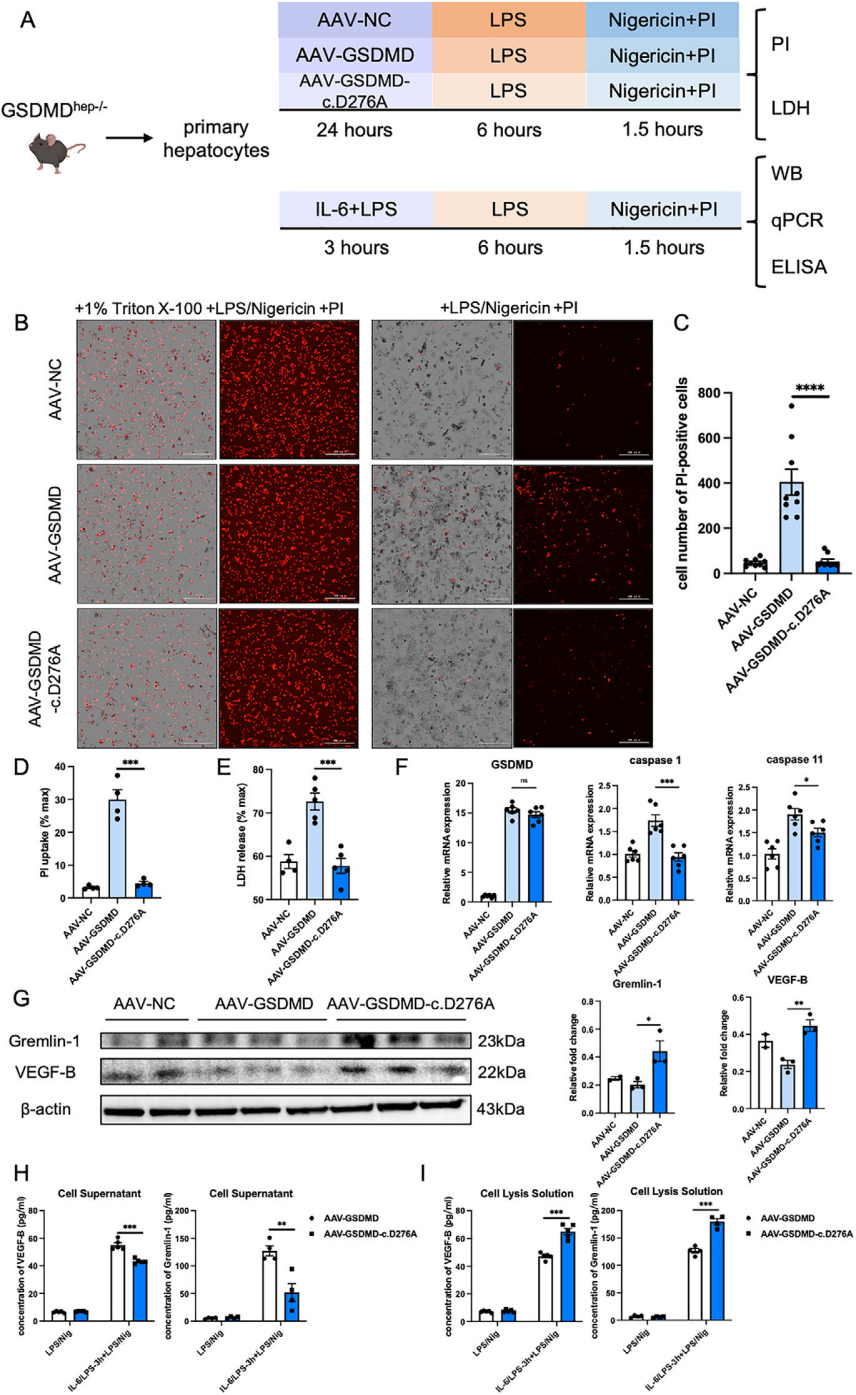
GSDMD induced the release of anti‐inflammatory factors in primary hepatocytes derived from GSDMD^hep‐/−^ mice through its pore‐forming activity. A) Schematic diagram of in vitro experiments on primary hepatocytes. B) PI staining in LPS/Nigericin‐treated primary hepatocytes derived from GSDMD^hep‐/−^ mice transfected with AAV‐NC, AAV‐GSDMD, and AAV‐GSDMD‐c.D276A. C,D) Cell number of PI‐positive cells (*n* = 9) and PI uptake (*n* = 4) in Figure A. E) LDH release of LPS/Nigericin‐treated primary hepatocytes derived from GSDMD^hep‐/−^ mice transfected with AAV‐NC, AAV‐GSDMD and AAV‐GSDMD‐c.D276A (*n* = 5). F) mRNA levels of GSDMD, caspase‐1 and caspase‐11 in LPS/Nigericin‐treated primary hepatocytes derived from GSDMD^hep‐/−^ mice transfected with AAV‐NC, AAV‐GSDMD and AAV‐GSDMD‐c.D276A (*n* = 6). G) Protein levels of Gremlin‐1 and VEGF‐B in LPS/Nigericin‐treated primary hepatocytes derived from GSDMD^hep‐/−^ mice transfected with AAV‐NC, AAV‐GSDMD and AAV‐GSDMD‐c.D276A (*n* = 3). H) Concentration of cell culture supernatant VEGF‐B and Gremlin‐1 in LPS/Nigericin‐treated primary hepatocytes derived from GSDMD^hep‐/−^ mice transfected with AAV‐GSDMD and AAV‐GSDMD‐c.D276A (*n* = 4–5). I) Concentration of cell lysis solution VEGF‐B and Gremlin‐1 in LPS/Nigericin‐treated primary hepatocytes derived from GSDMD^hep‐/−^ mice transfected with AAV‐GSDMD and AAV‐GSDMD‐c.D276A (*n* = 4–5). Data are presented as the mean ± SEM, ^*^ *p* <0.05, ^**^ *p* <0.01, ^***^ *p* <0.001, ^****^ *p* <0.001.

To further verify the role of GSDMD's pore‐forming activity in sepsis, we constructed full‐length GSDMD and point mutant GSDMD (c.D276A) plasmids and packaged them using adeno‐associated virus (AAV). We first treated primary hepatocytes derived from GSDMD^hep‐/−^ mice with these two AAVs, and the PI staining and LDH release analysis demonstrated that AAV‐GSDMD‐c.D276A inhibited the pore‐forming activity of GSDMD (**Figure** **6**B–E), as well as the pyroptosis of primary hepatocytes (Figure 6F,G). After the same IL‐6/LPS stimulation as above, the protein expression and ELISA results confirmed that the pore‐forming activity of GSDMD promoted the release of Gremlin‐1 and VEGF‐B from hepatocytes in sepsis (Figure 6H,I). Therefore, GSDMD might induce the release of anti‐inflammatory factors in primary hepatocytes through its pore‐forming activity.

### 2.6 Hepatocyte‐Specific Replenishment of GSDMD Alleviates Severe Systemic Inflammatory Reactions to LPS‐Induced Sepsis in GSDMD^hep‐/−^ Mice

**Figure 7:**
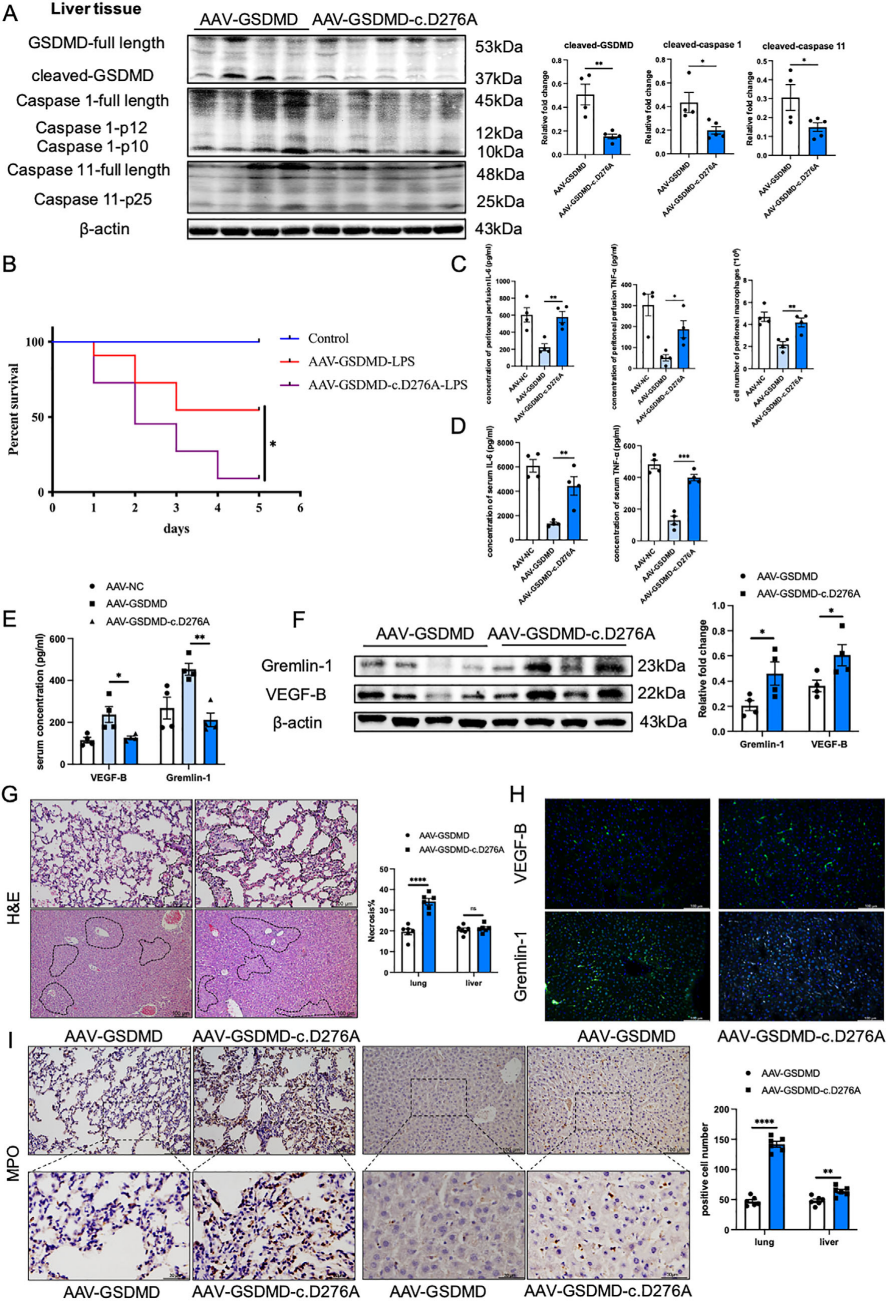
Hepatocyte‐specific replenishment of GSDMD alleviated severe systemic inflammatory reactions to LPS‐induced sepsis in GSDMD^hep‐/−^ mice. A) Protein levels of pyroptosis‐related proteins and their spliceosomes in LPS‐induced sepsis models of GSDMD^hep‐/−^ mice injected with AAV‐GSDMD and AAV‐GSDMD‐c.D276A (*n* = 4–5). B) Survival rate of mice in LPS‐induced sepsis models of GSDMD^hep‐/−^ mice injected with AAV‐GSDMD and AAV‐GSDMD‐c.D276A (*n* = 10). C) Contents of inflammatory factors in peritoneal perfusion of LPS‐induced sepsis models of GSDMD^hep‐/−^ mice injected with AAV‐GSDMD and AAV‐GSDMD‐c.D276A (*n* = 4). D) Contents of inflammatory factors in the serum of LPS‐induced sepsis models of GSDMD^hep‐/−^ mice injected with AAV‐GSDMD and AAV‐GSDMD‐c.D276A (*n* = 4). E) Contents of Gremlin‐1 and VEGF‐B in the serum of LPS‐induced sepsis models of GSDMD^hep‐/−^ mice injected with AAV‐GSDMD and AAV‐GSDMD‐c.D276A (*n* = 4). F) Protein levels of Gremlin‐1 and VEGF‐B in the liver tissues of LPS‐induced sepsis models of GSDMD^hep‐/−^ mice injected with AAV‐GSDMD and AAV‐GSDMD‐c.D276A (*n* = 4). G) H&E staining of lung and liver tissues in LPS‐induced sepsis models of GSDMD^hep‐/−^ mice injected with AAV‐GSDMD and AAV‐GSDMD‐c.D276A (*n* = 6). H) Immunofluorescent staining of Gremlin‐1 and VEGF‐B in the liver tissues of LPS‐induced sepsis models of GSDMD^hep‐/−^ mice injected with AAV‐GSDMD and AAV‐GSDMD‐c.D276A (Blue: DAPI, Green: VEGF‐B; Blue: DAPI, Green: HNF4α, Red: Gremlin‐1). I) MPO staining of lung and liver tissues in LPS‐induced sepsis models of GSDMD^hep‐/−^ mice injected with AAV‐GSDMD and AAV‐GSDMD‐c.D276A (*n* = 6). Data are presented as the mean ± SEM, ^*^p <0.05, ^**^p <0.01, ^***^p <0.001, ^****^ *p* <0.001.

We further explored the role of hepatocyte‐specific replenishment of GSDMD in LPS‐induced sepsis in GSDMD^hep‐/−^ mice. Four weeks after tail vein injection of AAV‐GSDMD and AAV‐GSDMD‐c.D276A, we found that hepatocyte‐specific replenishment of full‐length GSDMD could significantly improve the survival rate of mice (**Figure** **7**B). At the same time, compared with the mice injected with GSDMD‐c.D276A, which could not play a pore‐forming role, the levels of inflammatory factors in serum and peritoneal perfusion and the number of peritoneal macrophages in mice replenished with full‐length GSDMD were also significantly lower (Figure 7C,D), indicating that hepatocyte‐specific replenishment of full‐length GSDMD could effectively alleviate the inflammation response in mice with LPS‐induced sepsis. Similarly, the results of H&E staining and MPO staining of liver and lung tissues of the two groups of mice showed that hepatocyte‐specific replenishment of full‐length GSDMD could significantly reduce lung injury in septic mice, and could also reduce liver injury in septic mice to a certain extent (Figure 7G,I). In terms of anti‐inflammatory factors, the results of ELISA and western blotting showed that hepatocyte‐specific replenishment of full‐length GSDMD significantly increased the levels of VEGF‐B and Gremlin‐1 in the serum of septic mice, while significantly reduced the protein expression levels of VEGF‐B and Gremlin‐1 in the liver tissue of septic mice (Figure 7E,F), which was consistent with the results of immunofluorescence staining of liver tissue sections (Figure 7H). Therefore, we speculated that hepatocyte‐specific replenishment of full‐length GSDMD could release VEGF‐B and Gremlin‐1 in the mouse hepatocytes through pore‐forming activity, thereby alleviating the inflammatory response and lung injury in LPS‐induced septic mice.

### 2.7 Hepatic GSDMD, as Well as VEGF‐B and Gremlin‐1, Inhibit the Production of Inflammatory Factors from Peritoneal Macrophages

**Figure 8:**
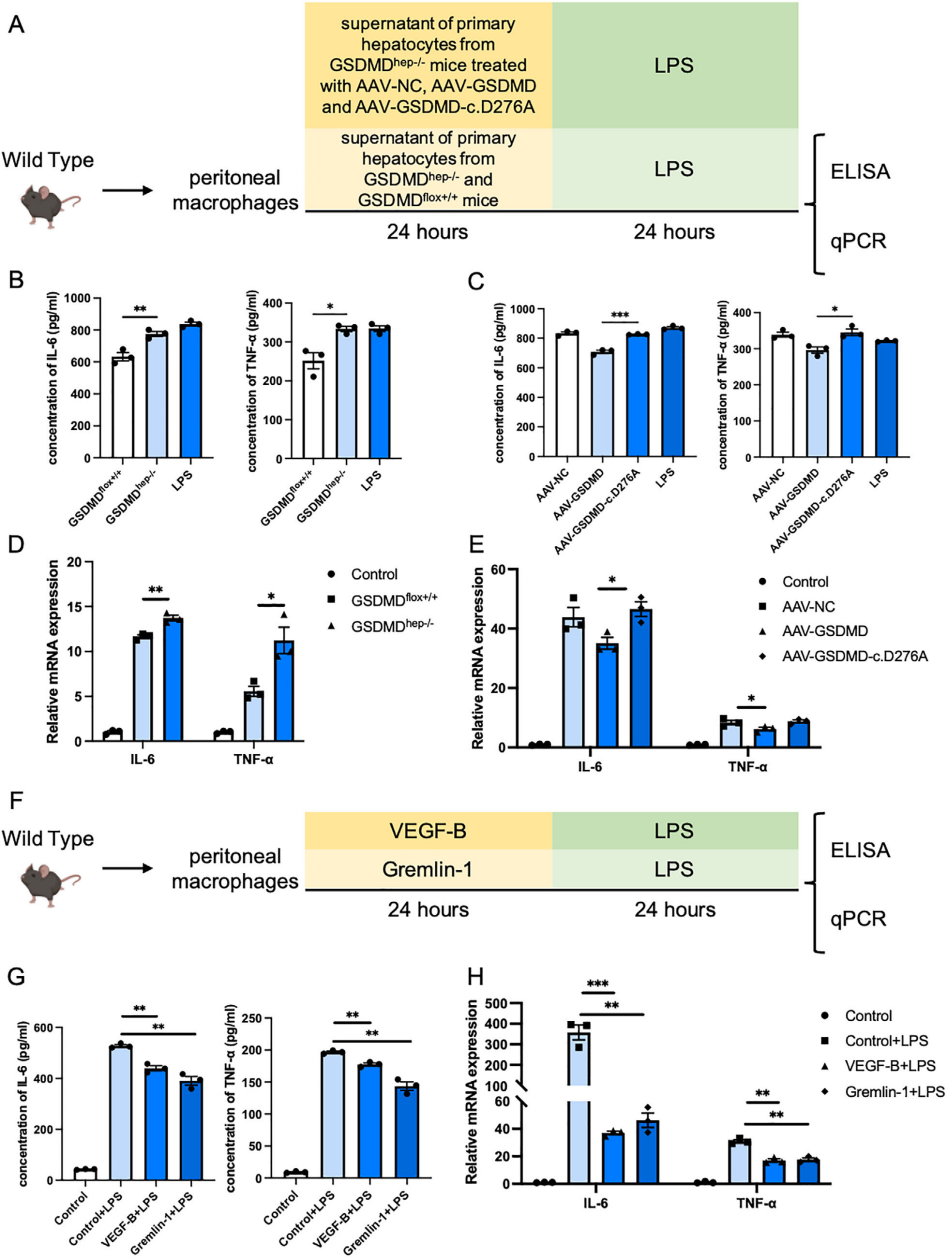
Hepatic GSDMD inhibited the production of inflammatory factors from peritoneal macrophages. A) Schematic diagram of in vitro experiments on peritoneal macrophages treated with different supernatant. B) Contents of inflammatory factors in the cell culture supernatant of LPS‐treated peritoneal macrophages co‐cultured with cell culture supernatant from primary hepatocytes derived from GSDMD^flox+/+^ mice and GSDMD^hep‐/−^ mice (*n* = 3). C) Contents of inflammatory factors in the cell culture supernatant of LPS‐treated peritoneal macrophages co‐cultured with cell culture supernatant from primary hepatocytes derived from GSDMD^hep‐/−^ mice transfected with AAV‐NC, AAV‐GSDMD and AAV‐GSDMD‐c.D276A (*n* = 3). D) mRNA levels of inflammatory factors of LPS‐treated peritoneal macrophages co‐cultured with cell culture supernatant from primary hepatocytes derived from GSDMD^flox+/+^ mice and GSDMD^hep‐/−^ mice (*n* = 3). E) mRNA levels of inflammatory factors of LPS‐treated peritoneal macrophages co‐cultured with cell culture supernatant from primary hepatocytes derived from GSDMD^hep‐/−^ mice transfected with AAV‐NC, AAV‐GSDMD and AAV‐GSDMD‐c.D276A (*n* = 3). F) Schematic diagram of in vitro experiments on peritoneal macrophages treated with VEGF‐B or Gremlin‐1. G) Contents of inflammatory factors in the cell culture supernatant of LPS‐treated peritoneal macrophages co‐cultured with VEGF‐B or Gremlin‐1 (*n* = 3). H) mRNA levels of inflammatory factors of LPS‐treated peritoneal macrophages co‐cultured with VEGF‐B or Gremlin‐1 (*n* = 3). Data are presented as the mean ± SEM, ^*^ *p* <0.05, ^**^ *p* <0.01, ^***^ *p* <0.001.

Finally, we explored how could hepatic GSDMD regulate systemic inflammatory responses in mice. Considering the important regulatory role of macrophages in inflammatory responses in sepsis, we extracted mouse peritoneal macrophages and co‐incubated them with supernatants of primary hepatocytes from GSDMD^flox+/+^ mice and GSDMD^hep‐/−^ mice, as well as supernatants of primary hepatocytes from GSDMD^hep‐/−^ mice injected with AAV‐GSDMD and AAV‐GSDMD‐c.D276A. Then, an in vitro pyroptosis model was established with LPS, and the production of inflammatory factors from the peritoneal macrophage was detected. The results showed that supernatants of primary hepatocytes from GSDMD^flox+/+^ mice and GSDMD^hep‐/−^ mice with hepatocyte‐specific replenishment of full‐length GSDMD could significantly inhibit the production of IL‐6 and TNF‐α from mouse peritoneal macrophages (**Figure** **8**B–E). Meanwhile, we also examined the role of VEGF‐B and Gremlin‐1 in this process. The results showed that treating mouse peritoneal macrophages with VEGF‐B or Gremlin‐1 alone could also inhibit the production of inflammatory factors (Figure 8G,H), indicating that hepatic GSDMD might release inflammatory inhibitory factors including VEGF‐B and Gremlin‐1 through pore‐forming activity to inhibit the production of inflammatory factors from macrophages, thereby alleviating the inflammatory response of mice with LPS‐induced sepsis.

## 3 Discussion and Conclusion

Pyroptosis is a mode of cell death regulated by dissolution and inflammation. Ion exchange inside and outside the cell membrane leads to increased intracellular osmotic pressure, cell swelling, and lysis. The signaling pathway mainly relies on inflammasomes to activate some proteins of the caspase family, causing them to cleave GSDM proteins, activate GSDM proteins, and translocate activated GSDM proteins to the membrane to form pores, finally leading to cell membrane rupture.^[^ ^16^ ^]^

A large number of studies have shown that pyroptosis plays an important role in the occurrence and development of sepsis and septic shock.^[^ ^17^, ^18^ ^]^ Pyroptosis dominates innate immune defense. And the loss of any link in the entire pathway from GSDMD to pyroptosis will lead to bacterial clearance disorders.^[^ ^19^ ^]^ As the executor of pyroptosis, GSDMD mediates the expression of different sepsis lesions and related molecules. Studies have shown that GSDMD can regulate coagulation disorders, oxidative stress, myocardial injury, liver injury, and intestinal injury in sepsis.^[^ ^18^, ^20^, ^21^, ^22^, ^23^ ^]^ In addition to hepatocyte‐specific knockout GSDMD mice, we also constructed myeloid‐specific knockout GSDMD mice (GSDMD^LysM‐/−^). Similar to the above results, the survival rate of GSDMD^LysM‐/−^ mice in LPS‐induced sepsis was significantly improved (Figure S2, Supporting Information).

However, with further in‐depth research on GSDMD, researchers have found that in addition to pyroptosis, GSDMD also plays a variety of other functions. For example, Karmakar et al. found that the cleaved N‐GSDMD produced during NLRP3 inflammasome signaling in neutrophils does not localize to the plasma membrane or increase plasma membrane permeability or cell pyroptosis, but instead secretes IL‐1β through an autophagy‐dependent mechanism.^[^ ^24^ ^]^ The formation of GSDMD pores was also initially identified as mediating the release of inflammatory factors such as IL‐1β and IL‐18, leading to cell death. However, cytokine release and cell death are two separate events.^[^ ^25^ ^]^ Evidence suggests that Ninjurin1, rather than GSDMD, is the direct cause of complete membrane disruption and cell death downstream of GSDMD pore formation.^[^ ^26^ ^]^ In the non‐canonical pathway, GSDMD pores formed after caspase‐4/5 activation can serve as a conduit for K^+^ efflux, leading to the formation of the NLRP3 inflammasome and caspase‐1‐dependent pro‐IL‐1β maturation.^[^ ^27^ ^]^ The passage of Ca^2+^ through the GSDMD pore can also activate membrane repair mechanisms, thereby rescuing cells from death.^[^ ^28^ ^]^ In sepsis, Liu et al. found that when GSDMD was depleted only in neutrophils, the severity of sepsis in mice was aggravated.^[^ ^29^ ^]^ Similarly, we demonstrated that the specific depletion of GSDMD in hepatocytes also aggravated sepsis in mice. Therefore, we further studied GSDMD in hepatocytes to explore whether it plays other roles besides cell pyroptosis.

In this study, we demonstrated in vitro and in vivo that hepatic GSDMD could release anti‐inflammatory factors through pore‐forming activity to inhibit the production of inflammatory factors by macrophages, thereby alleviating the inflammatory response in septic mice. Immune response dysregulation is a key factor in the pathogenesis of sepsis. Macrophages are considered to be the most important innate immune cells and antigen‐presenting cells, playing a vital role in regulating immune balance and inflammatory response.^[^ ^30^, ^31^ ^]^ Macrophage activation and its polarization state directly determine the immune status of sepsis patients to a certain extent.^[^ ^32^ ^]^ During sepsis, macrophages exhibit remarkable plasticity, allowing them to differentiate into different phenotypes, namely classically activated macrophages (M1 macrophages) and alternatively activated macrophages (M2 macrophages) in response to microenvironmental cues.^[^ ^33^ ^]^ M1 macrophages are proinflammatory and are responsible for initiating inflammatory responses necessary to control infection. However, excessive M1 activation can lead to uncontrolled inflammation and tissue damage.^[^ ^34^ ^]^ During the vigorous inflammatory response phase of sepsis, targeted inhibition of M1 macrophages can significantly reduce the release of inflammatory factors, thereby reducing tissue damage and patient mortality. Activated macrophages release a large number of proinflammatory cytokines (TNF‐α, IL‐1β, and IL‐6) and chemokines (CCL2, CCL5), leading to the recruitment of neutrophils and enhancing the inflammatory response.^[^ ^35^ ^]^ Targeted inhibition of M1 macrophage polarization can reduce tissue inflammatory infiltration, improve the survival rate of septic mice, and improve multiple organ damage caused by sepsis.^[^ ^36^, ^37^, ^38^, ^39^ ^]^

Based on serum proteomic screening, we found that VEGF‐B and Gremlin‐1 might be important anti‐inflammatory factors released by hepatic GSDMD. Vascular endothelial growth factor‐B (VEGF‐B) was first discovered in 1996 and has been proven to play a role in coordinating the crosstalk between angiogenesis and metabolism.^[^ ^40^, ^41^ ^]^ VEGF‐B can regulate vascular and cardiomyocyte growth, tissue metabolism, and cell survival in the heart, also playing an important role in the central nervous system and tissue protection.^[^ ^42^ ^]^ Gremlin‐1 is a 184‐amino acid protein with a cysteine knot motif and a cysteine‐rich region.^[^ ^43^ ^]^ Gremlin‐1 was originally identified as an antagonist of bone morphogenetic proteins 2, 4, and 7, which was mainly involved in organogenesis and limb patterning.^[^ ^44^ ^]^ However, Gremlin‐1 was subsequently shown to bind to VEGF‐R2, SLIT, macrophage migration inhibitory factor, and FGFR1, leading to changes in signal transduction through these pathways,^[^ ^45^, ^46^, ^47^ ^]^ playing a key role in tumors and various fibrotic diseases.^[^ ^48^, ^49^ ^]^ The specific role of VEGF‐B and Gremlin‐1 in sepsis is still unclear, but studies have confirmed that VEGF‐B and Gremlin‐1 play an important role in macrophage polarization. Li et al. found that Resveratrol could inhibit the M1 polarization of macrophages through the VEGFB/AMPK/NF‐кB pathway and had a potential therapeutic effect on myocardial injury. In this process, the M2‐promoting effect was specifically mediated by the VEGF‐B pathway.^[^ ^50^ ^]^ Mthunzi et al. confirmed that Gremlin‐1 was required for macrophages to undergo M2 polarization in response to Th2 cytokines IL‐4 and IL‐13. Exogenous Gremlin‐1 could enhance macrophage polarization, characterized by increased expression of the profibrotic M2 polarization markers Arg1 and Fizz1.^[^ ^51^ ^]^

This study proposes for the first time that liver‐specific GSDMD plays a protective role in sepsis, but it still has certain limitations. Further in‐depth research is needed on the role of the screened VEGF‐B and Gremlin‐1 in sepsis.

## 4 Experimental Section

### Animals

Male WT C57BL/6 mice (6–8 weeks old) were purchased from Shanghai Jiesijie Laboratory Animal Co.Ltd. (Shanghai, China). GSDMD^flox+/+^ mice were designed by GemPharmatech Co.Ltd. GSDMD^flox+/+^ mice were crossed with Alb‐Cre+ mice (purchased from Shanghai Biomodel Organism Science and Technology Development Co. Ltd.) to generate hepatocyte‐specific GSDMD knockout mice (GSDMD^hep‐/−^). In all experiments, GSDMD^flox+/+^ littermates were used as controls. All mouse strains had a C57BL/6J background and were housed in plastic cages under a 12:12‐h light/dark photoperiod at controlled temperature with free access to water and food. All animal procedures were conducted according to the Guide for the Care and Use of Laboratory Animals and were approved by the Animal Care and Use Committee of Shuguang Hospital Affiliated to Shanghai University of Traditional Chinese Medicine (approval number: PZSHUTCM2412170002).

### LPS Model

Mice were intraperitoneally injected with LPS (Sigma–Aldrich, #L2630, MO, USA) 5 or 20 mg kg^−1^ (for survival rate experiments) to induce sepsis, and samples were collected 6 h after the model was established for subsequent experiments.

### CLP Model

After the mouse was completely anesthetized, small scissors were used to cut the outer layer of the mouse's abdominal fur, then the peritoneum was cut to expose the abdominal cavity. Small tweezers were used to gently reach into the abdominal cavity to find the cecum end, the cecum end was removed and ligated according to the experimental needs (for survival rate experiments, 2/3 of the cecum end should be ligated; for non‐survival rate experiments, 1/2 of the cecum end should be ligated). After ligation, the needle tip of a 5 mL syringe was used to perforate the cecum at the ligated site. Then the ligated and perforated cecum were put back into the mouse body, and the mouse peritoneum and outer layer of fur were sutured with surgical sutures. Samples were collected 16 h after the model was established for subsequent experiments.

### ALT/AST

Blood was collected from the aorta of each mouse and centrifuged at 3000 rpm for 10 min, and the concentrations of alanine aminotransferase (ALT) and aspartate aminotransferase (AST) were measured using an ALT/AST kit (Nanjing Jiancheng Bioengineering Institute, Nanjing, China).

### H&E Staining

Lung tissue and liver tissue were fixed in 4% paraformaldehyde for 24 h and then embedded in paraffin. Paraffin sections were cut into 5 µm and H&E stained using a hematoxylin and eosin staining kit (Beyotime Biotechnology, Shanghai, China).

### Immunohistochemical (IHC) Staining

Lung tissue and liver tissue were fixed in 4% paraformaldehyde for 24 h and then embedded in paraffin. Paraffin sections were cut into 5 µm, and paraffin sections were heated at 65 °C for 5 min, immersed in xylene twice for 5 min each time, 100% alcohol twice for 3 min each time, 95% alcohol twice for 3 min each time, ddH2O for 5 min, and microwave‐fixed with citric acid for 15 min. Then, the sections were washed with PBS (MeilunBio, Dalian, China) three times for 3 min each time, blocked with 10% BSA (Sangon Biotech, Shanghai, China) for 1 h, and incubated with MPO (DSHB, #CPTC‐MPO‐1), GSDMD (abcam, #ab209845, Cambridge, UK), VEGF‐B (R&D Systems, #MAB751, MN, USA), Gremlin‐1 (CST, #4383, MA, USA) and other primary antibodies at 4 °C overnight. On the second day, the sections were taken out from 4 °C and equilibrated at room temperature for 20 min, washed with PBS three times for 5 min each time, incubated with secondary antibody (1% BSA dilution) at room temperature for 1 h, washed with PBS three times for 5 min each time, developed with DAB, rinsed with running water for 10 min, counterstained with hematoxylin for 35 s, rinsed with running water for 10 min, 95% alcohol twice for 10 s each time, 100% alcohol twice for 10 s each time, xylene twice for 10 s each time, and sealed with neutral gum.

### Immunofluorescent (IF) Staining

Liver tissue was fixed in 4% paraformaldehyde for 24 h and then embedded in paraffin. Paraffin sections were cut into 5 µm, and paraffin sections were heated at 65 °C for 5 min, immersed in xylene twice for 5 min each time, 100% alcohol twice for 3 min each time, 95% alcohol twice for 3 min each time, ddH2O for 5 min, and microwave‐fixed with citric acid for 15 min. After being washed with PBS three times for 3 min each time, the sections were blocked with 10% BSA for 1 h and incubated with VEGF‐B (R&D Systems, #MAB751, MN, USA) and Gremlin‐1 (CST, #4383, MA, USA) antibodies at 4 °C overnight. On the second day, the sections were taken out from 4 °C and balanced at room temperature in the dark for 20 min, washed with PBS three times for 5 min each time, incubated with the corresponding fluorescent secondary antibody (diluted with 1% BSA) at room temperature in the dark for 1 h, washed with PBS three times for 5 min each time, and stained with DAPI for nuclei and sealed.

### Quantitative Real‐Time Polymerase Chain Reaction (qPCR)

Total RNA was purified from ≈30 mg liver samples or cell lysates according to the manufacturer's protocol (ShareBio, Shanghai, China), and then 500 ng mRNA was reverse transcribed to cDNA using a HiScript II Q RT SuperMix for qPCR (Vazyme Biotech, Nanjing, China). Relative quantitative gene expression was measured via real‐time PCR using a ViiATM 7 Real‐Time PCR System (Life Technologies) and a ChamQ Universal SYBR qPCR Master Mix (Vazyme Biotech, Nanjing, China). GAPDH expression was used as an internal standard. The primer sequences are as follows:

**Table:** 

Gene	Sense	Anti‐sense
GAPDH	AGGCCGGTGCTGAGTATGTC	TGCCTGCTTCACCACCTTCT
GSDMD	CCATCGGCCTTTGAGAAAGTG	ACACATGAATAACGGGGTTTCC
caspase‐1	ACAAGGCACGGGACCTATG	TCCCAGTCAGTCCTGGAAATG
caspase‐11	ACAAACACCCTGACAAACCAC	CACTGCGTTCAGCATTGTTAAA
IL‐1β	GCAACTGTTCCTGAACTCAACT	ATCTTTTGGGGTCCGTCAACT
IL‐6	TGTTCTCTGGGAAATCGTGGA	TTTCTGCAAGTGCATCATCGT
TNF‐α	TTCTATGGCCCAGACCCTCA	TTTGCTACGACGTGGGCTAC
Activin A	TCACCATCCGTCTATTTCAGCA	CTTCCGAGCATCAACTACTTTCT
Gremlin‐1	ATCATCAACCGCTTCTGTTATGG	AGTCGATGGATATGCAACGGC
VEGF‐B	GCCAGACAGGGTTGCCATAC	GGAGTGGGATGGATGATGTCAG
sFRP‐3	CACAGCACCCAGGCTAACG	TGCGTACATTGCACAGAGGAA
Progranulin	ATGTGGGTCCTGATGAGCTG	GCTCGTTATTCTAGGCCATGTG
Arg‐1	CTCTGTTCAGCTATTGGACGC	CGGAATTTCTGGGATTCAGCTTC
CD206	CTCTGTTCAGCTATTGGACGC	CGGAATTTCTGGGATTCAGCTTC
INOS	CTCCAAGCCAAAGTCCTTAGAG	AGGAGCTGTCATTAGGGACATC
Mrc‐1	CTCTGTTCAGCTATTGGACGC	CGGAATTTCTGGGATTCAGCTTC

### Western Blotting

After the liver tissue was homogenized with RIPA lysis buffer (Beyotime Biotechnology, Shanghai, China), the supernatant was centrifuged at 13 000 rpm for 10 min, and Western blot was performed after BCA protein quantification. After preparing 10% gel, the gel was run at a constant voltage of 100V, followed by 400 mA transfer for 45 min. After being washed with TBST (Solarbio Life Sciences, Beijing, China) three times for 10 min each time, the membranes were blocked with 5% skimmed milk powder (Sangon Biotech, Shanghai, China) for 1 h. The membranes were incubated with GSDMD (abcam, #ab209845, Cambridge, UK), caspase‐1 (Santa Cruz Biotechnology, #sc‐56036, TX, USA), caspase‐11 (Santa Cruz Biotechnology, #sc‐374615, TX, USA), VEGF‐B (R&D Systems, #MAB751, MN, USA), Gremlin‐1 (CST, #4383, MA, USA) and other primary antibodies at 4 °C overnight. On the second day, the membranes were washed with TBST three times for 10 min each time, incubated with the corresponding secondary antibodies at room temperature for 1 h, and washed with TBST three times for 10 min each time. Finally, the proteins were visualized using an ECL Luminescence Reagent (MeilunBio, Dalian, China).

### Peritoneal Perfusion

After killing the mouse by cervical dislocation, small scissors were used to cut the abdominal. Then, 5 mL PBS was injected into the mouse's abdominal cavity, and then sucked out from the abdominal cavity using a syringe. Finally, the lavage fluid was centrifuged at 300g for 5 min, and the supernatant was collected after centrifugation for the subsequent experiments.

### Enzyme‐Linked Immunosorbent Assay (ELISA)

ELISA kits were used to determine IL‐6 (Invitrogen, CA, USA), TNF‐α. (Invitrogen, CA, USA), IL‐1β (Multi Sciences, Hangzhou, China), IL‐10 (Multi Sciences, Hangzhou, China), IL‐18 (Multi Sciences, Hangzhou, China), VEGF‐B (Teye Biotechnology, Shanghai, China) and Gremlin‐1 (Teye Biotechnology, Shanghai, China) levels in the serum, peritoneal perfusion, and cell supernatants according to the manufacturer's protocols.

### Isolation of Primary Hepatocytes

After anesthesia, the mouse was opened, and an intravenous indwelling needle was inserted into the mouse portal vein. First, 9.5 mg of EGTA (Sangon Biotech, Shanghai, China) diluted with 50 mL Hanks' Balanced Salt Solution (HBSS, Beyotime Biotechnology, Shanghai, China) was perfused to flush out the blood in the liver, and then 40 mL of Gey's Balanced Salt Solution (GBSS, Macgene, Beijing, China) containing 0.075% collagenase (Sigma–Aldrich, #C5138, MO, USA) was perfused for 5 min. After removing the liver, it was cut into pieces and digested in GBSS containing 0.008% collagenase at 37 °C for 15 min. Then, it was passed through a 70 µm cell sieve, centrifuged at 50g for 3 min, and the precipitate was taken. It was then washed twice with GBSS, and the precipitate (hepatocytes) was collected and counted.

### PI Staining

Mouse primary hepatocytes were stimulated with 1 µg mL^−1^ LPS for 6 h and then stimulated with 20 µm Nigericin (Selleck, #S6653, TX, USA) and 1 µg mL^−1^ PI dye (Sangon Biotech, #A601112, Shanghai, China) for 1.5 h. The red fluorescence ratio of cells was observed under a fluorescence microscope.

### LDH Release

After inducing pyroptosis model in mouse primary hepatocytes in vitro, the cell supernatant was collected, and cell perforation was detected using the LDH detection kit (Nanjing Jiancheng Bioengineering Institute, Nanjing, China).

### Serum Proteomics

GSDMD^flox+/+^ mice and GSDMD^hep‐/−^ mice were intraperitoneally injected with 5 mg kg^−1^ LPS to induce sepsis, and blood was collected from the orbits 6 h after membrane formation for serum proteomics analysis.

### Viral Vectors and Infection

Adeno‐associated virus 8 (AAV8) packaging full‐length GSDMD and mutant GSDMD plasmid (GSDMD‐c.D276A) that cannot be cleaved were constructed and packaged by Beijing Tsingke Biotech Co., Ltd. (Beijing, China). In vitro infection of primary hepatocytes and Aml12 cell lines with AAV was performed at a multiplicity of infection (MOI) of 1E4. Aml12 cell lines were cultured in DMEM/F12 medium (Thermo Fisher Scientific, MA, USA) with 10% Fetal bovine serum (FBS, Thermo Fisher Scientific, MA, USA).

### AAV Tail Vein Injection

GSDMD^hep‐/−^ mice were injected with AAV‐GSDMD and AAV‐GSDMD‐c.D276A viruses (10^12^ vg) through the tail vein, and sepsis models were induced with LPS and CLP 4 weeks later.

### Isolation of Mouse Peritoneal Macrophages

Three days after 3–4 mL of broth was injected into the mouse peritoneal cavity, the mouse was killed by cervical dislocation. Small scissors were used to cut the outer layer of the peritoneal fur. Then, 5 mL PBS was injected into the mouse's abdominal cavity, and then sucked out from the abdominal cavity using a syringe. Another 5 mL of PBS was used to wash the mouse peritoneal cavity to obtain more peritoneal macrophages. The cells were centrifuged at 1200 rpm for 10 min and then resuspended in RPMI 1640 medium (Thermo Fisher Scientific, MA, USA) with 10% Fetal bovine serum (FBS, Thermo Fisher Scientific, MA, USA).

### Statistical Analysis

All data were presented as the mean ± SEM of at least 3 biological replicates per group. Data were analyzed using Student's *t*‐tests for only 2 groups and one‐way ANOVA tests for multiple groups. Experimental results were analyzed using GraphPad Prism 9. *p*‐values <0.05 were considered statistically significant.

## Conflict of Interest

The authors declare no conflict of interest.

## Supporting information



Supporting Information

## Data Availability

The data that support the findings of this study are available on request from the corresponding author. The data are not publicly available due to privacy or ethical restrictions.
