# Supplementary material for: Hepatocyte‐Specific GSDMD Deficiency Aggravates Sepsis by Disrupting Non‐Canonical Secretion of Anti‐Inflammatory Factors
Source: Adv Sci (Weinh). 2025 Aug 26;12(43):e10412. doi: 10.1002/advs.202510412 (PMC12631855; doi:10.1002/advs.202510412)
Supplement: Supplementary file 1 — Supporting Information [file ADVS-12-e10412-s001.docx]

Supporting Information

Hepatic GSDMD alleviates sepsis by inhibiting macrophages inflammatory factors production via regulating the release of anti-inflammatory factors from hepatocytes

Yihan Qian, Bingrui, Wang, Chang Yu, Yuge Zhou, Weifan Huang, Xing Rong, Yali Sang, Jiangang Song*, Hailong Wu*, and Xiaoni Kong*

**Supplementary Figure 1. Generation and validation of GSDMD^hep-/-^ and GSDMD^flox+/+^ mice
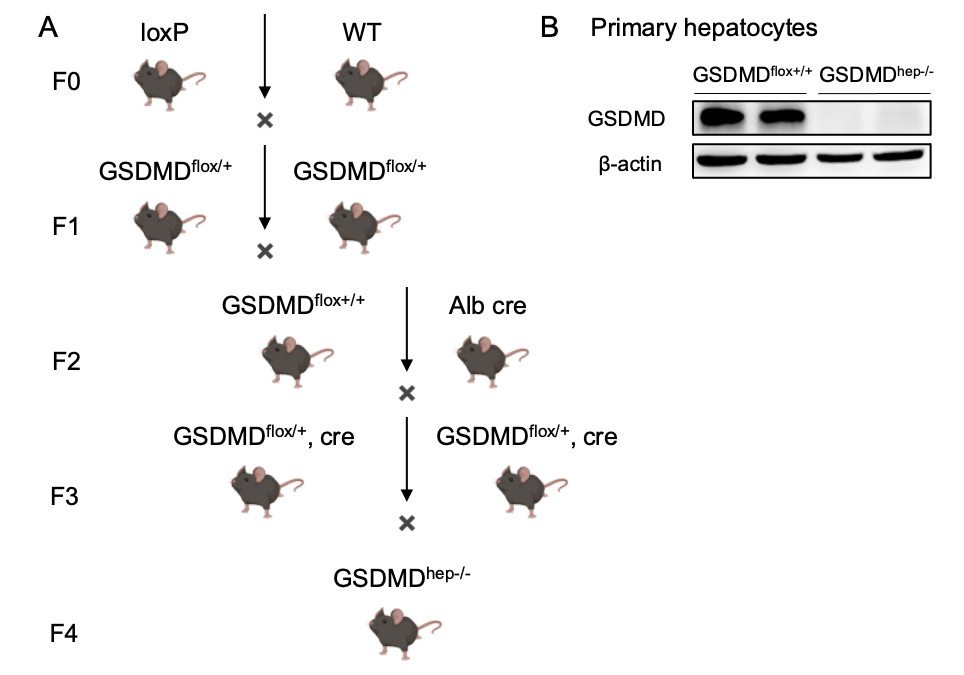
.**

A. Generation of GSDMD^hep-/-^ and GSDMD^flox+/+^ mice.

B. Protein levels of GSDMD in the liver of GSDMD^hep-/-^ and GSDMD^flox+/+^ mice.


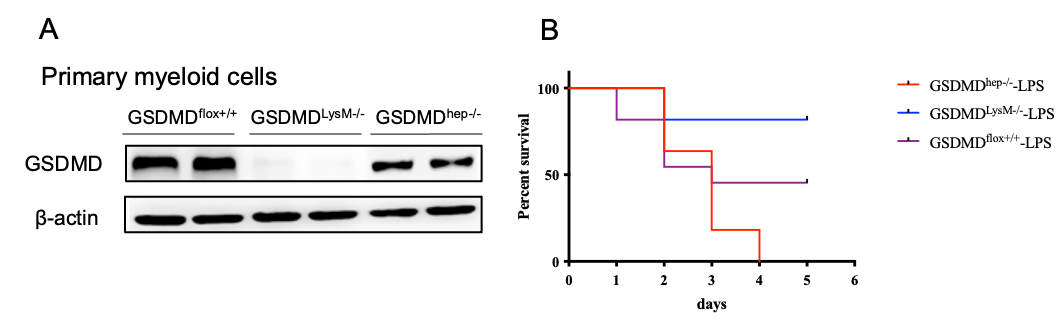
**Supplementary Figure 2. Myeloid-specific knockout of GSDMD exacerbates LPS-induced sepsis.**

A. Protein levels of GSDMD in the primary myeloid cells of GSDMD^hep-/-^, GSDMD^flox+/+^ and GSDMD^LysM-/-^ mice.

B. Survival rate of mice in LPS-induced sepsis models of GSDMD^hep-/-^, GSDMD^flox+/+^ and GSDMD^LysM-/-^ mice.

**
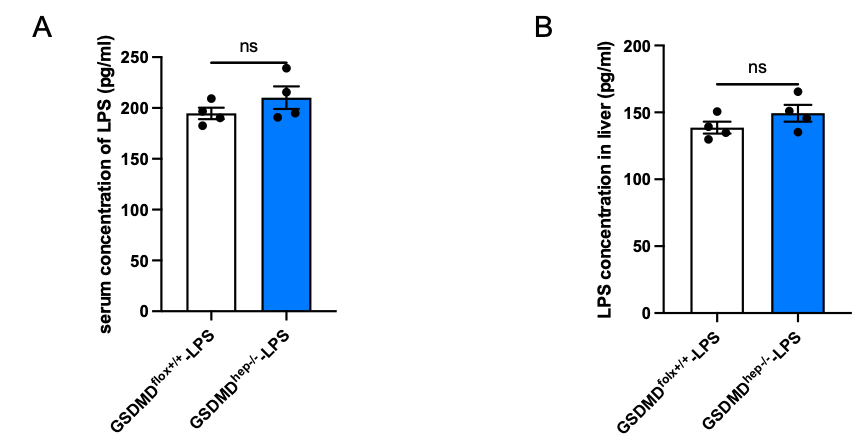
**

**Supplementary Figure 3. Hepatic GSDMD deficient does not affect the distribution or absorption of LPS.**

A. Contents of LPS in the serum of LPS-induced sepsis models of GSDMD^flox+/+^ mice and GSDMD^hep-/-^ mice (n = 4).

B. Contents of LPS in the liver of LPS-induced sepsis models of GSDMD^flox+/+^ mice and GSDMD^hep-/-^ mice (n = 4).


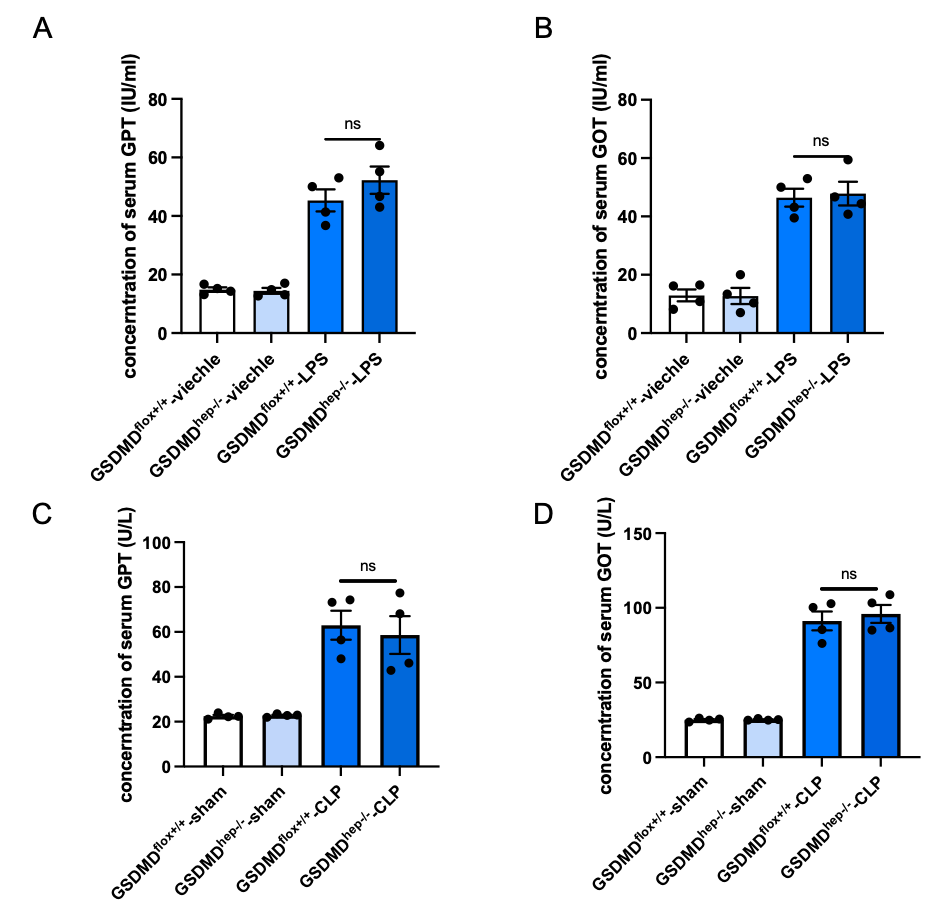
**Supplementary Figure 4. Hepatic GSDMD deficient does not affect the dysfunction of liver.**

A. Concentration of GPT in the liver of LPS-induced sepsis models of GSDMD^flox+/+^ mice and GSDMD^hep-/-^ mice (n = 4).

B. Concentration of GOT in the liver of LPS-induced sepsis models of GSDMD^flox+/+^ mice and GSDMD^hep-/-^ mice (n = 4).

C. Concentration of GPT in the liver of CLP-induced sepsis models of GSDMD^flox+/+^ mice and GSDMD^hep-/-^ mice (n = 4).

D. Concentration of GOT in the liver of CLP-induced sepsis models of GSDMD^flox+/+^ mice and GSDMD^hep-/-^ mice (n = 4).


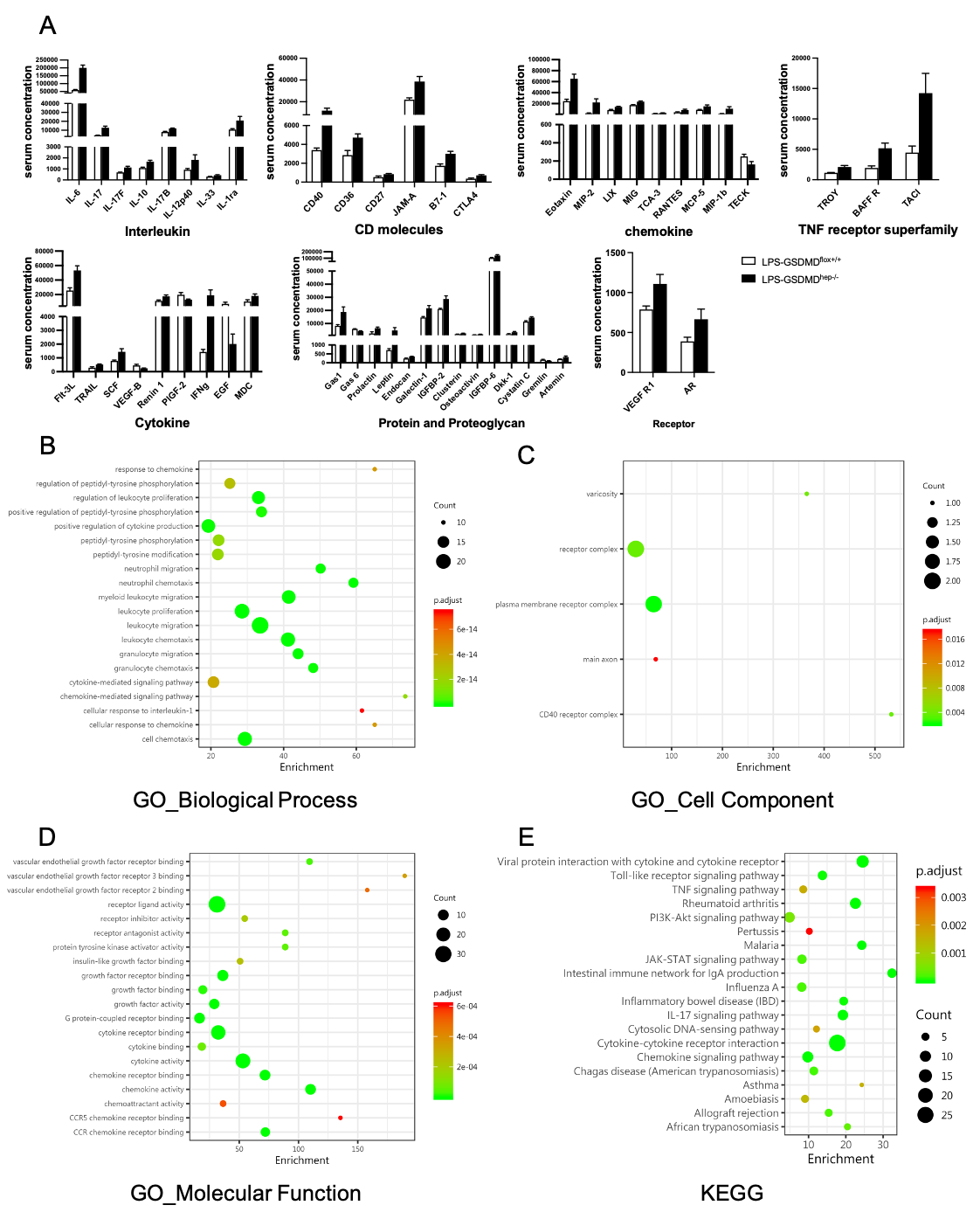
**Supplementary Figure 5. Analysis of serum proteomics.**

A. Contents of different types of inflammatory factors and anti-inflammatory factors in the serum of LPS-induced sepsis models of GSDMD^flox+/+^ mice and GSDMD^hep-/-^ mice.

B. GO analysis of LPS-treated GSDMD^flox+/+^ mice and GSDMD^hep-/-^ mice focusing on Biological Process.

C. GO analysis of LPS-treated GSDMD^flox+/+^ mice and GSDMD^hep-/-^ mice focusing on Cell Component.

D. GO analysis of LPS-treated GSDMD^flox+/+^ mice and GSDMD^hep-/-^ mice focusing on Molecular Function.

E. KEGG analysis of LPS-treated GSDMD^flox+/+^ mice and GSDMD^hep-/-^ mice.


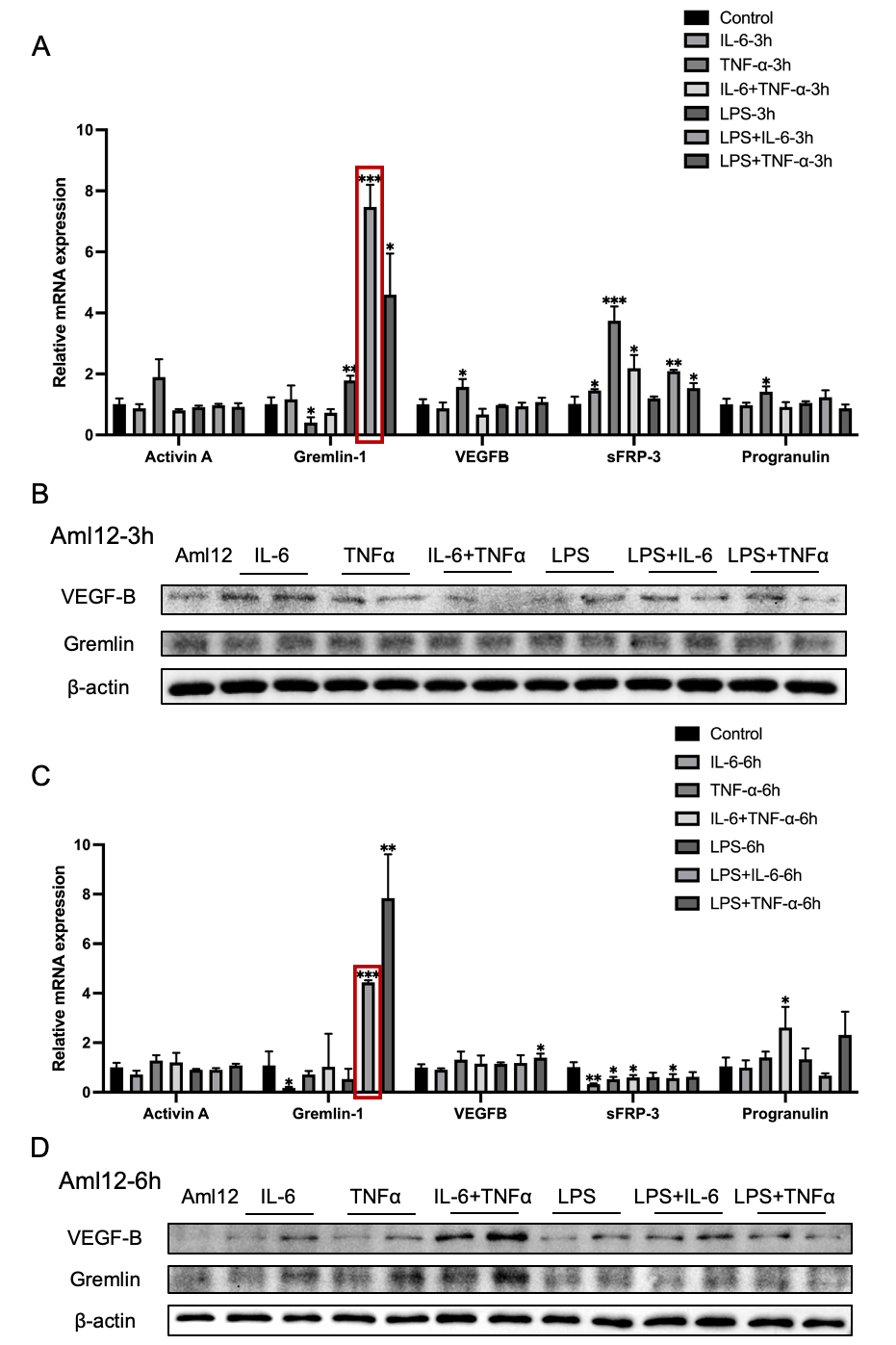
**Supplementary Figure 6. Effects of inflammatory factors on activating Aml12 cells lines to express anti-inflammatory factors.**

A. mRNA levels of Activin A, Gremlin-1, VEGF-B, sFRP-3 and Progranulin in the *in vitro* pyroptosis model of Aml12 cell lines after 3 hours treatment of different inflammatory factors.

B. Protein levels of Gremlin-1 and VEGF-B in the *in vitro* pyroptosis model of Aml12 cell lines after 3 hours treatment of different inflammatory factors.

C. mRNA levels of Activin A, Gremlin-1, VEGF-B, sFRP-3 and Progranulin in the *in vitro* pyroptosis model of Aml12 cell lines after 6 hours treatment of different inflammatory factors.

D. Protein levels of Gremlin-1 and VEGF-B in the *in vitro* pyroptosis model of Aml12 cell lines after 6 hours treatment of different inflammatory factors.


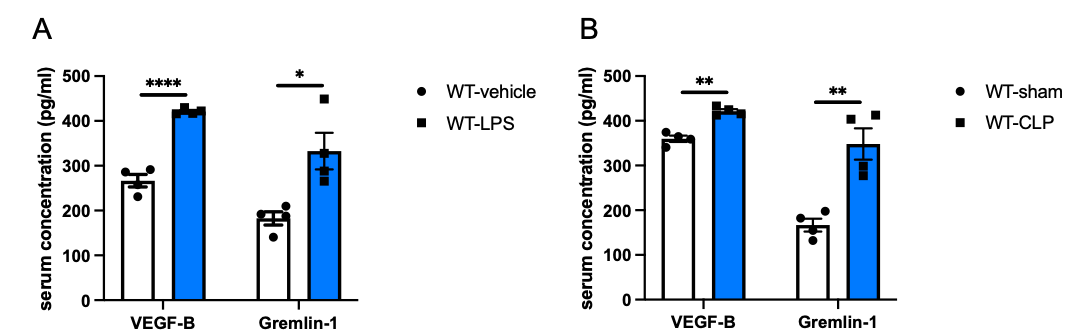
**Supplementary Figure 7. Serum concentration of VEGF-B and Gremlin-1 increases in sepsis model.**

A. Serum concentration of VEGF-B and Gremlin-1 in LPS model (n = 4, **P* < 0.05, *****P* < 0.0001)

B. Serum concentration of VEGF-B and Gremlin-1 in CLP model (n = 4, ***P* < 0.01)

**
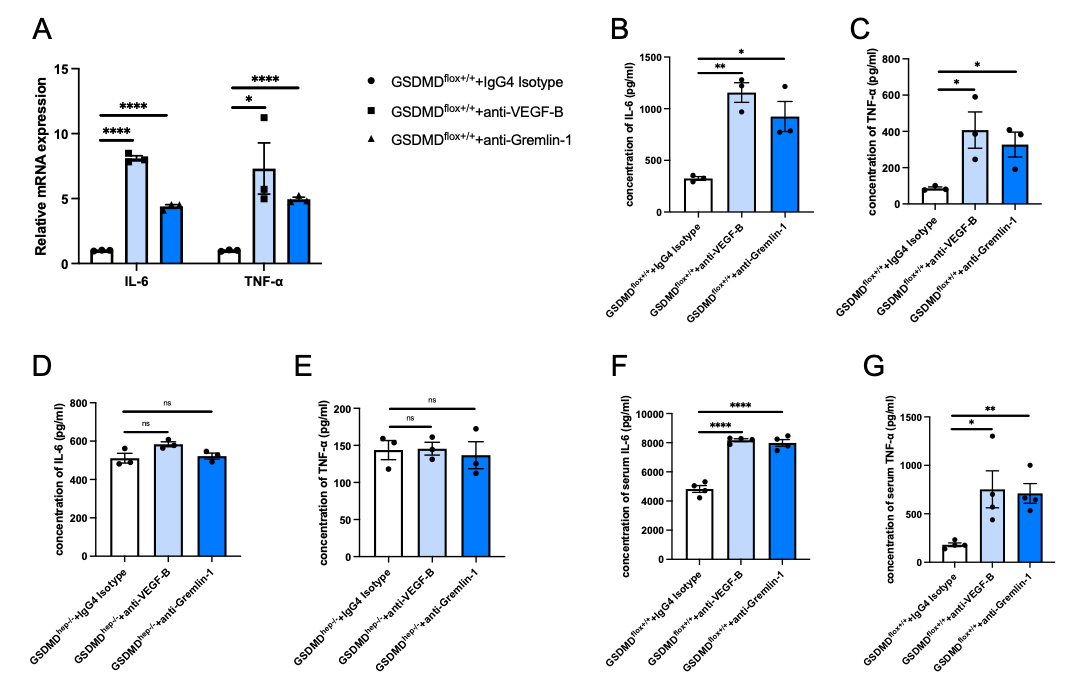
Supplementary Figure 8. Neutralization of VEGF-B and Gremlin-1 abrogates the anti-inflammatory effect of GSDMD.**

A. mRNA levels of IL-6 and TNF-α of LPS-treated peritoneal macrophages co-cultured with cell culture supernatant from primary hepatocytes derived from GSDMD^flox+/+^ and VEGF-B and Gremlin-1 neutralizing antibodies (n = 3, **P* < 0.05, *****P* < 0.0001).

B. Contents of IL-6 in the cell culture supernatant of LPS-treated peritoneal macrophages co-cultured with cell culture supernatant from primary hepatocytes derived from GSDMD^flox+/+^ and VEGF-B and Gremlin-1 neutralizing antibodies (n = 3, **P* < 0.05, ***P* < 0.01).

C. Contents of TNF-α in the cell culture supernatant of LPS-treated peritoneal macrophages co-cultured with cell culture supernatant from primary hepatocytes derived from GSDMD^flox+/+^ and VEGF-B and Gremlin-1 neutralizing antibodies (n = 3, **P* < 0.05).

D. Contents of IL-6 in the cell culture supernatant of LPS-treated peritoneal macrophages co-cultured with cell culture supernatant from primary hepatocytes derived from GSDMD^hep-/-^ and VEGF-B and Gremlin-1 neutralizing antibodies (n = 3).

E. Contents of TNF-α in the cell culture supernatant of LPS-treated peritoneal macrophages co-cultured with cell culture supernatant from primary hepatocytes derived from GSDMD^hep-/-^ and VEGF-B and Gremlin-1 neutralizing antibodies (n = 3).

F. Serum concentration of IL-6 in LPS-induced sepsis models of GSDMD^flox+/+^ mice treated with VEGF-B and Gremlin-1 neutralizing antibodies (n = 4, *****P* < 0.0001)

G. Serum concentration of TNF-α in LPS-induced sepsis models of GSDMD^flox+/+^ mice treated with VEGF-B and Gremlin-1 neutralizing antibodies (n = 4, **P* < 0.05, ***P* < 0.01)


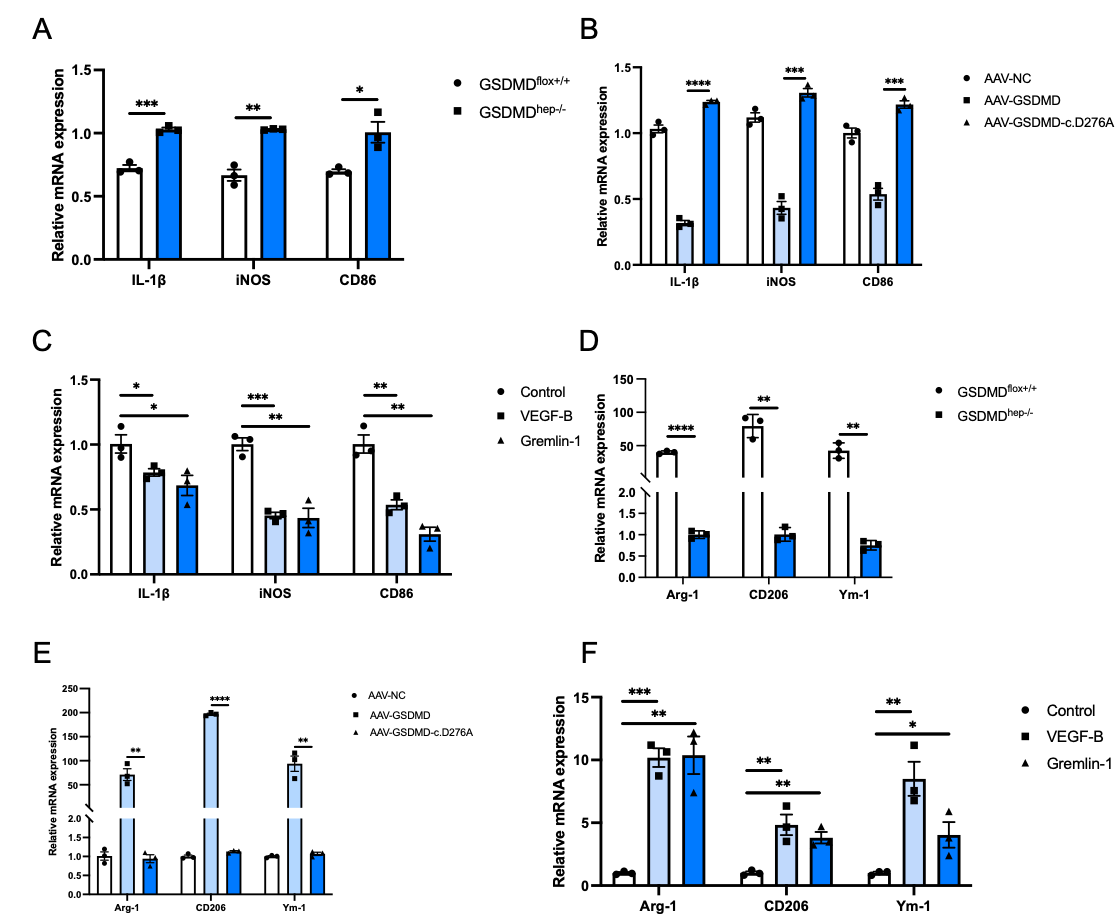
**Supplementary Figure 9. Increased VEGF-B and Gremlin-1 in primary hepatocytes from GSDMD^hep-/-^ mice play a M2 polarization-inducing role.**

A. mRNA levels of M1 markers (IL-1β, iNOS, CD86) of LPS-treated peritoneal macrophages co-cultured with cell culture supernatant from primary hepatocytes derived from GSDMD^flox+/+^ mice and GSDMD^hep-/-^ mice (n = 3, **P* < 0.05, ***P* < 0.01, ****P* < 0.001).

B. mRNA levels of M1 markers (IL-1β, iNOS, CD86) of LPS-treated peritoneal macrophages co-cultured with cell culture supernatant from primary hepatocytes derived from GSDMD^hep-/-^ mice transfected with AAV-NC, AAV-GSDMD and AAV-GSDMD-c.D276A (n = 3, ****P* < 0.001, *****P* < 0.0001).

C. mRNA levels of M1 markers (IL-1β, iNOS, CD86) of LPS-treated peritoneal macrophages co-cultured with cell culture supernatant from primary hepatocytes derived from GSDMD^hep-/-^ mice treated with with recombinant VEGF-B and Gremlin-1 (n = 3, **P* < 0.05, ***P* < 0.01, ****P* < 0.001).

D. mRNA levels of M2 markers (Arg-1, CD206, Ym-1) of LPS-treated peritoneal macrophages co-cultured with cell culture supernatant from primary hepatocytes derived from GSDMD^flox+/+^ mice and GSDMD^hep-/-^ mice (n = 3, ***P* < 0.01, *****P* < 0.0001).

E. mRNA levels of M2 markers (Arg-1, CD206, Ym-1) of LPS-treated peritoneal macrophages co-cultured with cell culture supernatant from primary hepatocytes derived from GSDMD^hep-/-^ mice transfected with AAV-NC, AAV-GSDMD and AAV-GSDMD-c.D276A (n = 3, ***P* < 0.01, *****P* < 0.0001).

F. mRNA levels of M2 markers (Arg-1, CD206, Ym-1) of LPS-treated peritoneal macrophages co-cultured with cell culture supernatant from primary hepatocytes derived from GSDMD^hep-/-^ mice treated with with recombinant VEGF-B and Gremlin-1 (n = 3, **P* < 0.05, ***P* < 0.01, ****P* < 0.001).
